# Restoration of gamma-aminobutyric acid homeostasis: A novel approach to alleviating central nervous system injury–associated immunodepression syndrome

**DOI:** 10.4103/NRR.NRR-D-25-00147

**Published:** 2025-09-03

**Authors:** Ping Yang, Di Tian, Zijiao Li, Zhongxiang Yao

**Affiliations:** 1 Department of Neurobiology, Army Medical University, Chongqing, China; 2 Cadet Brigade 4, College of Basic Medicine, Army Medical University, Chongqing, China; 3 Department of Physiology, Army Medical University, Chongqing, China

**Keywords:** activity-dependent rehabilitation, central nervous system injury–associated immunodepression syndrome, dietary interventions, excitatory-inhibitory balance, gamma-aminobutyric acid, microglial modulation, nerve regeneration, potassium -chloride cotransporter 2, spinal cord injury, sympathetic hyperreflexia

## Abstract

Injuries to the central nervous system can disrupt body functions and often cause excessive sympathetic activity, leading to immune suppression known as central nervous system injury–associated immunodepression syndrome. The connection between central nervous system injury and central nervous system injury–associated immunodepression syndrome is not fully clear. Gamma-aminobutyric acid, an important inhibitory neurotransmitter, helps excitation-inhibition balance in the nervous system, especially after spinal cord injuries. Impaired gamma-aminobutyric acid signaling causes an excitation-inhibition imbalance, which worsens neural plasticity, increases sympathetic overactivity, and may lead to central nervous system injury–associated immunodepression syndrome. This review discusses the roles of gamma-aminobutyric acid in protecting central nervous system structure and function and how its dysfunction contributes to abnormal plasticity and heightened reflexes. We also explore new treatments aimed at restoring gamma-aminobutyric acid balance, such as modulating potassium-chloride cotransporter 2, enhancing activity-dependent recovery, targeting microglial responses, and dietary approaches. Maintaining healthy gamma-aminobutyric acid activity is essential for preventing immune issues following central nervous system injury. This review emphasizes the regulation of gamma-aminobutyric acid as a promising target for future treatments of central nervous system injury-associated immunodepression syndrome.

## Introduction

Maintaining a proper balance between excitatory and inhibitory signaling within the central nervous system (CNS) is essential for controlling movement patterns, promoting neural plasticity, and regulating immune responses (Brennan et al., 2024). Multiple inhibitory processes continually shape this balance, and the gamma-aminobutyric acid (GABA) system plays a central role in regulating neuronal excitability and maintaining stable neural networks (Frankowski et al., 2022; Hudson and Grau, 2022; Abusaada et al., 2025). Disruptions to GABA signaling can have widespread effects on CNS function. There is evidence that changes in GABA activity are closely linked to neural dynamics in various critical neurological disorders across different life stages (Kim and Yoon, 2017; Krueger-Burg, 2025; Perovic et al., 2025; Tao et al., 2025). The inhibitory function of GABA is crucial in preventing hyperexcitability and maladaptive plasticity resulting from excitation-inhibition imbalances (Frankowski et al., 2022; Lam et al., 2023; Saraswathy et al., 2024; Krueger-Burg, 2025; Perovic et al., 2025).

Dysfunction in GABA signaling has been associated with various neurological conditions, such as traumatic brain injury, stroke, epilepsy, depression, substance use disorders, and neurodevelopmental disorders like schizophrenia, Rett syndrome, autism spectrum disorder, and spinal cord injury (Tang, 2020; Frankowski et al., 2022; Krueger-Burg, 2025; Perovic et al., 2025). These abnormalities often contribute to impairments in cognition, motor control, and immune regulation, highlighting the critical role of the GABA system in maintaining CNS homeostasis.

CNS injury–associated immunodepression syndrome (CIDS) is a serious complication of CNS injury that causes immune suppression and raises the risk of infection, such as pneumonia (Członkowska et al., 1979; Jackson and Groomes, 1994). This infection affects up to 22% of stroke patients (Meisel et al., 2005). Infections also contribute to death after spinal cord injury (DeVivo et al., 1993; Jackson and Groomes, 1994; Burkovskiy et al., 2016; Rodgers et al., 2022; Sribnick et al., 2022; DiSabato et al., 2024). CIDS results from disrupted neuroimmunomodulation pathways, mainly involving sympathetic preganglionic neurons in the brain and spinal cord. These neurons regulate the autonomic and immune systems. This network includes the cortex, thalamus, hypothalamus, reticular formation, hippocampus, cerebellum, brainstem, and spinal cord. These structures work together to maintain balance in the body (Meisel et al., 2005; Brennan et al., 2021, 2024; Talifu et al., 2022). Spinal cord injury damages connections from the brainstem to sympathetic preganglionic neurons, impairing normal sympathetic reflexes. This leads to overactive responses, or hyperreflexia, and immune suppression, as demonstrated by numerous studies (Lucin et al., 2007; Zhang et al., 2013; Ueno et al., 2016; Prüss et al., 2017; Brennan et al., 2021, 2024; Jeffries and Tom, 2021; Noble et al., 2022; Talifu et al., 2022).

This review focuses on how GABA maintains stable CNS function and how GABA-related issues contribute to conditions such as CIDS. It discusses the cellular and molecular changes that occur after brain injury, stroke, spinal cord injury, and other CNS issues. Particular attention is given to how abnormal plasticity leads to sympathetic overreaction and immune suppression. Additionally, the review explores new treatment strategies, including adjusting the activity of the potassium (K^+^)-chloride (Cl^–^) cotransporter 2 (KCC2), neural activity-based rehabilitation, modulation of microglia, and dietary interventions. These strategies aim to restore GABA function and reduce the effects of CIDS. A better understanding of the link between GABA, neural plasticity, and the immune response could lead to more effective CIDS management strategies.

## Search Strategy

This review used PubMed to find studies on immune suppression associated with CNS injury or disease. Several key search terms were used: “immune dysfunction driven by CNS disorders” (1378 papers from January 1966 to June 2025); “GABAergic system in CNS disorders” (506 papers from January 1997 to June 2025); “immune dysfunction and GABAergic system” (168 papers from January 1984 to June 2025). Only articles in English were included for consistency.

Out of these, 317 studies were chosen for their relevance to immune decline after traumatic brain injury, stroke, spinal cord injury, and other CNS issues, especially how the sympathetic nervous system is affected. These papers were reviewed to understand how GABA issues develop and how restoring GABA might help treat CIDS.

**Figure 1:**
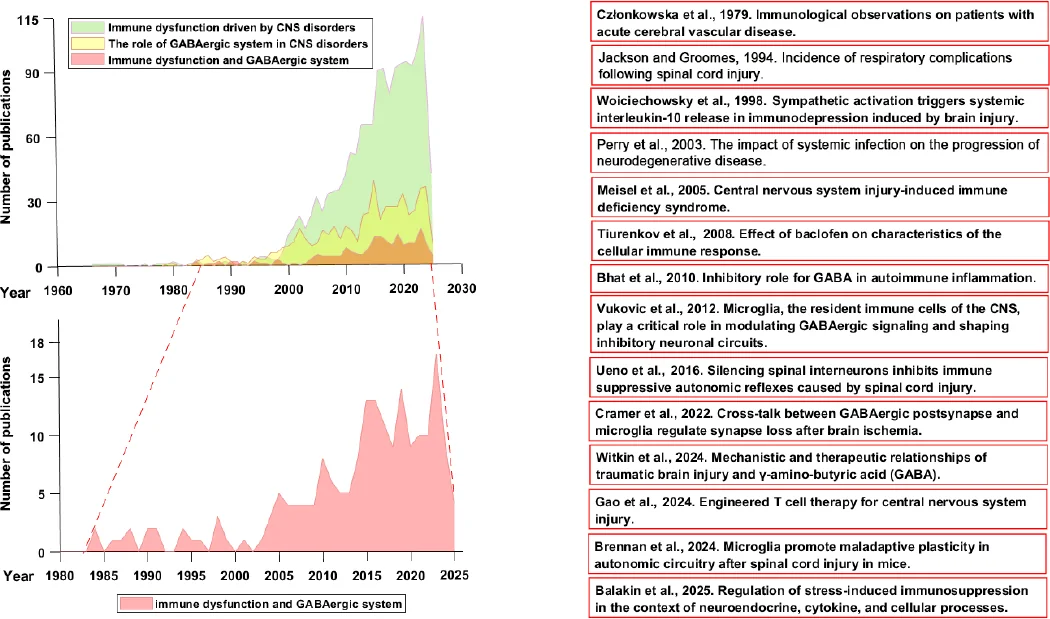
Landmark studies of the connection between immune dysfunction and the GABA system in CNS disorders. These seminal investigations (representing initial discoveries across specialized fields) elucidate the progression of mechanistic insights into how gamma-aminobutyric acid signaling underpins immune dysregulation. They shed light on mechanisms underlying CIDS, highlighting how GABA deficits following CNS trauma can contribute to immunodepressive states. It shows that the number of related publications is increasing over the past 20 years, as reflected in PubMed data. Created with MATLAB R2022a. CIDS: Central nervous system injury–associated immunodepression syndrome; CNS: central nervous system; GABA: gamma-aminobutyric acid.

Important studies that deepen our understanding of the role of GABA in CIDS and recovery are highlighted. They are listed in order of publication and focus on how GABA maintains stable CNS function and how GABA dysfunction contributes to conditions like CIDS (**Figure 1**). The goal of combining these findings is to improve the understanding of the role of GABA and guide new treatments for CIDS.

## Potential Mechanisms behind Central Nervous System Injury–associated Immunodepression Syndrome

Following CNS injuries, infections can exacerbate neurodegeneration and impede the recovery process. This underscores the importance of understanding the underlying mechanisms of CIDS (Perry et al., 2003; Failli et al., 2012). Understanding these pathways is essential for developing targeted treatments to address immune dysfunction following CNS trauma.

### Maladaptive neuroplasticity

One complex aspect of CIDS involves maladaptive changes in the brain and spinal cord. Abnormal growth of excitatory interneurons occurs alongside a reduction in GABA-mediated inhibition (Brennan et al., 2024; Gillespie et al., 2024). This imbalance amplifies sympathetic reflex circuits, resulting in increased sympathetic activity and immune response suppression (Meisel et al., 2005; Zhang et al., 2013; Brennan et al., 2021, 2024). These findings are clinically significant; however, our current therapy options are limited because we lack a comprehensive understanding of how GABA systems malfunction after injury and contribute to altered plasticity and immune suppression.

#### Structural disruptions in sympathetic neural pathways

Injuries to the CNS, such as traumatic brain injury and spinal cord injury, often result in widespread immune disturbances, commonly known as CIDS (Burkovskiy et al., 2016; Sribnick et al., 2022). Current research suggests that CIDS arises from a complex network of mechanisms, including the release of pathogen-associated molecular patterns, the expansion of myeloid-derived suppressor cells, and autonomic nervous system dysregulation. Following acute CNS trauma, damaged tissues release damage-associated molecular patterns that trigger inflammatory responses. The initial inflammation is often followed by systemic immunodepression. Meanwhile, myeloid-derived suppressor cells, which were first characterized in tumor immunology as immunosuppressive myeloid cells, are recruited and play a role in dampening immune activity (Woiciechowsky et al., 2002; Sribnick et al., 2022; Dobson et al., 2024).

A key factor in the development of CIDS is dysregulation of the autonomic nervous system, particularly a shift toward sympathetic dominance following severe trauma. This heightened sympathetic activity is often accompanied by decreased parasympathetic function and interferes with neuroimmune signaling, destabilizing systemic balance (Tracey, 2002; Pavlov and Tracey, 2004). Damage to CNS components that regulate the autonomic nervous system significantly contributes to the immunodepression observed in CIDS.

Emerging research highlights the influence of sympathetic nervous system activation on immune cell behavior. For instance, animal models of traumatic brain injury have revealed a link between increased sympathetic nervous system activation and elevated levels of programmed cell death-1 on CD4^+^ and CD8^+^ T cells. This fosters an immunodepressive environment (Yang et al., 2019). Similarly, excessive activation of sympathetic reflex pathways is a defining feature of CIDS (Lucin et al., 2007; Zhang et al., 2013; Ueno et al., 2016; Prüss et al., 2017; Mironets et al., 2020; Noble et al., 2022; Brennan et al., 2024).

The sympathetic neural substrates involved in neuromodulation are located within a critical network of brain and spinal regions. This network encompasses higher-order cortical regions, such as the frontal premotor cortex; subcortical nuclei, such as the thalamus and hypothalamus; and the brainstem, which contains the reticular formation. Additionally, the hippocampus plays a role in memory and the stress response, and the cerebellum is responsible for motor coordination. Within the spinal cord, the lateral column and intermedial nuclei are of particular interest. These regions are essential for autonomic and immune functions and are highly vulnerable to injury-associated disturbances (Talifu et al., 2022; Brennan et al., 2024).

The sympathetic nervous system plays a crucial role in immune regulation by extensively innervating key lymphoid structures, including the thymus, bone marrow, spleen, lymph nodes, and mucosa-associated lymphoid tissues. When injury occurs in the CNS, the normal pre-ganglionic sympathetic pathways may become disrupted. This disruption can result in abnormal reflex activity within these organs. These abnormal sympathetic responses often lead to the overproduction and release of catecholamines from nerve endings, which further suppresses immune function (Meisel et al., 2005).

Increased sympathetic activity following injury can negatively impact organ performance, particularly blood flow to the intestines. This can lead to gut ischemia, increased intestinal permeability, and the passage of bacterial components, such as lipopolysaccharides, into the bloodstream. These bacterial products can intensify systemic inflammation and boost pathogen-associated molecular patterns, which worsens immune dysfunction (Sudo, 2014; Mayer et al., 2015; Dobson et al., 2024).

Clinical studies support the idea that excessive sympathetic activation contributes to immune suppression (Jeffries and Tom, 2021; Brennan et al., 2024). For instance, in trauma patients, blocking the sympathetic nervous system, particularly with drugs targeting β-adrenergic receptors, has been associated with reduced levels of inflammatory cytokines, including tumor necrosis factor α (TNF-α), interleukin (IL)-1β, and IL-6 (Ni Choileain and Redmond, 2006; Liu et al., 2013). Conversely, levels of the anti-inflammatory cytokine IL-10 tend to rise during periods of heightened sympathetic activity following traumatic brain injury. Early research suggests that using β-adrenergic receptor blockers, such as propranolol, can reduce IL-10 surges in a dose-dependent manner, indicating a potential strategy for restoring immune balance by modulating sympathetic responses (Woiciechowsky et al., 1998; Bouras et al., 2022).

**Figure 2:**
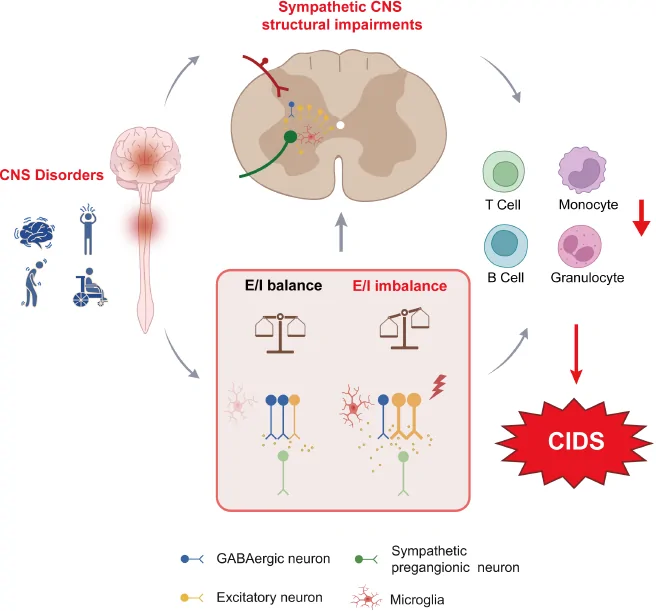
Hypothesized pathways in CIDS. Traumatic events affecting the CNS produce structural modifications within the sympathetic nervous system, disrupt GABA transmission, and activate microglia. These processes are interconnected: alterations in GABA signaling influence microglial activity, which subsequently impacts neuroinflammation, GABA metabolism, and synaptic plasticity. Interruptions in inhibitory GABA circuits lead to an excitation–inhibition imbalance in neural networks, which worsens structural damage and impairs immune organ function. Collectively, these changes may drive the development of CIDS, characterized by immunodepression following trauma. Created with Adobe illustrator 2020. CIDS: Central nervous system injury-associated immunodepression syndrome; CNS: central nervous system; E/I: excitatory–inhibitory; GABA: gamma-aminobutyric acid.

These findings underscore the critical role of the sympathetic nervous system in the brain and spinal cord in the development of CIDS. When the sympathetic nervous system becomes dysregulated after CNS trauma, communication between the nervous and immune systems is impaired, leading to immunodepression and a higher risk of infection (**Figure 2**). Understanding how sympathetic hyperactivation influences immune function is essential for developing targeted treatments to lessen CIDS and improve patient recovery.

#### Disruption of the excitatory–inhibitory neurotransmitter balance

GABA signaling is essential for mediating inhibitory synaptic transmission and for developing and maturing inhibitory synapses and axons. GABA signaling plays a crucial role in refining neural circuits by facilitating the elimination of redundant nascent synaptic connections (Wu et al., 2012). Similar to glutamate at excitatory synapses, GABA coordinates the maturation of presynaptic and postsynaptic elements within inhibitory synapses. This promotes activity-dependent synapse formation and refinement (Huang, 2009; Wu et al., 2012). CNS injuries, such as traumatic brain injury and spinal cord injury, disrupt GABA signaling. This results in the inadequate formation and maturation of inhibitory synapses, as well as increased network excitability. These factors contribute to sympathetic hyperreflexia and systemic immunodepression.

Additionally, the loss of GABAergic interneurons and inhibitory synapses increases connectivity between excitatory interneurons and sympathetic preganglionic or motor neurons, exacerbating conditions such as immunodepression, spasticity, and autonomic dysreflexia (Ueno et al., 2016; Gong et al., 2021; Mazzone et al., 2021; Brennan et al., 2024). Prophylactic interventions such as the GABA analog gabapentin, when administered immediately after injury, have shown promise in reducing the formation of excitatory synapses and subsequently lowering the risk of epileptogenesis (Prince et al., 2016).

The GABA system comprises GABAergic interneurons, GABA receptors, GABA-synthesizing enzymes (glutamic acid decarboxylase [GAD] isoforms GAD-65 and GAD-67), the vesicular GABA transporter, and KCC2. This system is crucial for maintaining the structural and functional integrity of the CNS. This system plays a key role in regulating the balance between excitation and inhibition, neural plasticity, and neuroimmune interactions. This highlights its importance in both physiological and pathological contexts (Zhang et al., 2021). Disruptions within the system following CNS injuries can contribute to the development of CIDS, underscoring the importance of developing targeted therapeutic strategies aimed at restoring GABA function to improve outcomes in affected individuals.

*Gamma-aminobutyric acid synthesis, degradation, and re-uptake*

GABA is the primary inhibitory neurotransmitter in the CNS (Guan et al., 2025). It is produced through the decarboxylation of glutamate, a process catalyzed by the enzyme GAD. Glutamate originates via transamination of α-ketoglutarate, a key intermediate in cellular energy metabolism within the tricarboxylic acid cycle. This process involves phosphate-activated glutaminase, which highlights the intricate relationship between metabolism and neurotransmitter production (Martin and Rimvall, 1993).

GABA synthesis begins with its packaging into synaptic vesicles. This process is mediated by the vesicular GABA transporter and depends on maintaining electrochemical gradients and proper pH within the vesicles. This ensures that GABA is readily available for release during synaptic transmission (McIntire et al., 1997; Chaudhry et al., 1998). Once released into the synaptic cleft, GABA is rapidly taken up by nearby neurons and astrocytes via high-affinity GABA transporters. This process not only recycles GABA back into the vesicles for subsequent use but also prevents excessive receptor activation and helps keep inhibitory signaling tightly controlled (Eulenburg and Gomeza, 2010).

GABA is primarily broken down by enzymes such as GABA transaminase and succinic semialdehyde dehydrogenase. These enzymes convert GABA into succinate. Succinate then enters the Krebs cycle, producing energy to support neuron function (Bak et al., 2006; Guerriero et al., 2015).

GABA levels depend on astrocytes near synapses. These cells absorb GABA released at synapses and convert it into succinate using GABA transaminase and succinic semialdehyde dehydrogenase. The succinate can then be used to produce glutamine, which is taken up by neurons and converted into glutamate. This process demonstrates the collaboration between astrocytes and neurons, particularly in maintaining inhibitory signals via the glutamate-glutamine cycle (Bak et al., 2006; Schousboe et al., 2013).

The levels of GABA outside of cells are primarily regulated by GABA transporters. These transporters reabsorb GABA from the synaptic cleft, which ends the inhibitory signal and preserves the balance between excitation and inhibition. Unlike other neurotransmitters, which break down enzymatically outside of cells, GABA is primarily regulated by reuptake (Sears and Hewett, 2021).

GABA production and reuptake are precisely regulated processes that are essential for neural homeostasis. Blocking these processes, as occurs in traumatic brain injury, weakens inhibitory signals and may lead to CIDS. Injury-related energy deficits, such as poor blood flow and metabolic issues, reduce GABA synthesis by disrupting the Krebs cycle (Yoshino et al., 1991; Guerriero et al., 2015). Altered GABA transporter activity has been linked to epilepsy, traumatic brain injury, and neurodegeneration. For example, lower levels of GABA transporter 3 in thalamic astrocytes are associated with neurological issues following traumatic brain injury. Increasing GABA transporter 3 levels can reduce seizures and improve survival, demonstrating therapeutic potential (Cho et al., 2022; Witkin et al., 2024).

A better understanding of GABA metabolism could lead to therapies that restore inhibition and reduce post-injury neural damage.

*Gamma-aminobutyric acid and other neurotransmitters*

GABA strongly interacts with glutamate and glutamine cycles. The cycle maintains the excitation-inhibition balance by exchanging precursor molecules between neurons and glia. It provides the key substrate required for neurotransmitter synthesis, highlighting how GABA and glutamate collectively regulate brain activity (Bak et al., 2006).

Glutamate, the principal excitatory neurotransmitter, is synthesized from α-ketoglutarate, an intermediate in the tricarboxylic acid cycle, within glutamatergic neurons (Huang et al., 2024a). Upon neuronal activation, glutamate is released into the synaptic cleft, where it binds to postsynaptic receptors to mediate excitatory signaling. Synaptic glutamate is quickly taken up by astrocytes, where it is converted to glutamine by the glutamine synthetase. The non-toxic precursor is then released into the extracellular space and can be absorbed by both glutamatergic and GABAergic neurons (Bak et al., 2006).

In GABAergic neurons, glutamine is converted back into glutamate via glutaminase. Glutamate then becomes GABA through the enzyme GAD (Bak et al., 2006). Mammals mainly have two GAD isoforms: GAD-65 and GAD-67 (Mazzone et al., 2021). GAD-65 is mainly at axon terminals, supporting activity-dependent GABA synthesis, while GAD-67 is spread across the cell body and dendrites, maintaining baseline GABA levels (Tillakaratne et al., 2000). Both isoforms allow flexible control of GABA production, ensuring effective inhibition.

After synthesis, GABA is packaged into vesicles by vesicular GABA transporter proteins and released into the synapse during calcium-triggered exocytosis (Bak et al., 2006). GABA then binds to postsynaptic GABA_A_ receptors (GABA_A_Rs) and GABA_B_ receptors (GABA_B_Rs) to produce inhibitory signals. It is quickly cleared by high-affinity GABA transporters on neurons and astrocytes, terminating inhibition and recycling GABA (Bormann, 2000; Perovic et al., 2025).

**Figure 3:**
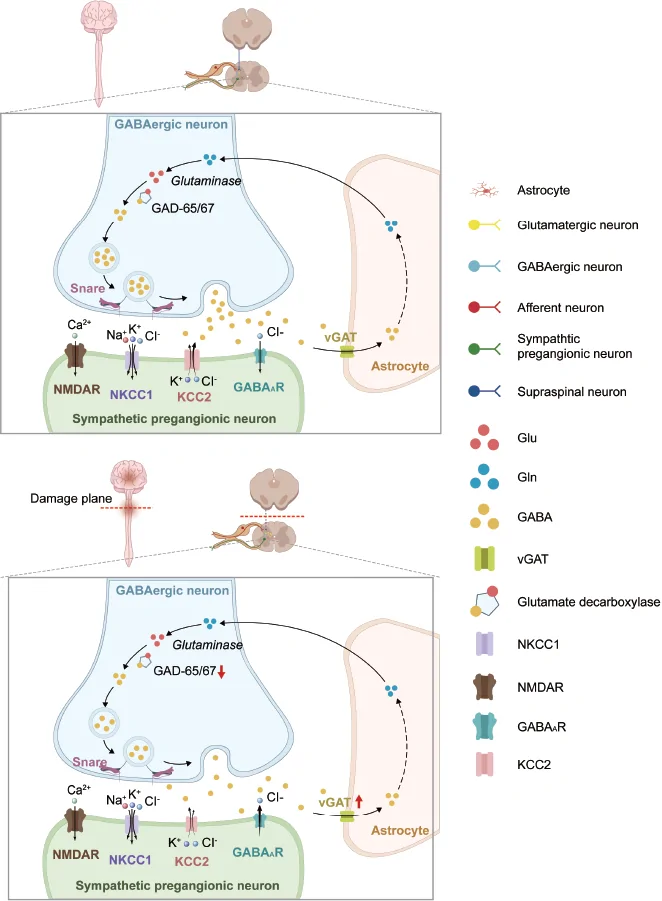
Consequences of GABA system disruption after spinal cord injury. The GABAergic system, including interneurons that release GABA, GABA_A_Rs, key enzymes GAD-65 or GAD-67 responsible for GABA synthesis, vGAT, and KCC2, remains central to controlling the spinal sympathetic reflex circuit. Within the intermediolateral region, GABAergic interneurons located near sympathetic preganglionic neurons significantly influence the activity of sympathetic preganglionic neurons, modulating spinal sympathetic reflex circuit responses. The efficacy of GABA’s inhibitory effect relies heavily on the maintenance of proper intracellular chloride ionic concentration, mainly regulated by KCC2. The interplay among KCC2, NKCC1, and other components of the GABA system determines neural function under both normal and pathological states. After spinal cord injury, various molecular changes, such as the loss of GABAergic interneurons, decreased GABA levels, downregulation of GAD-65 or GAD-67, reduced KCC2 expression, increased activity of GABA reuptake transporter, and disrupted chloride ionic homeostasis, contribute to the emergence of spinal cord injury-associated immunodepression syndrome. These molecular disruptions impair spinal plasticity, worsen the excitation-inhibition imbalance, and compromise overall neural integrity. Created with Adobe illustrator 2020. Ca^2+^: Calcium ion; Cl^–^: chloride ion; CNS: central nervous system; E/I: excitatory-inhibitory; GABA: gamma-aminobutyric acid; GABA_A_R: GABAA receptor; GAD: glutamic acid decarboxylase; Glu: glutamate; Gln: glutamine; K^+^: potassium; KCC2: potassium (K^+^)-chloride (Cl^–^) cotransporter 2; Na^+^: sodium ion; NKCC1: Na^+^-K^+^-2Cl^–^-cotransporter 1; NMDAR: N-methyl-D-aspartate receptor; vGAT: vesicular GABA transporter.

The glutamate-GABA-glutamine cycle supports ongoing neurotransmitter synthesis and reflects the metabolic partnership between neurons and astrocytes. Astrocytes convert glutamate into glutamine, preventing excitotoxicity and providing GABA precursors (**Figure 3**). The metabolic control is vital for maintaining excitatory-inhibitory balance, which is essential for healthy brain function (Andersen et al., 2022). Disruptions, such as traumatic brain injury or neurodegeneration, impair the cycle and cause neurological problems.

Understanding the interactions between GABA and glutamate within the cycle offers insights into the complex mechanisms underlying neural communication. It also points toward potential therapeutic strategies aimed at restoring the excitatory-inhibitory balance critical for normal brain activity.

*Gamma-aminobutyric acid receptor subtypes and their functions*

GABA serves as the primary inhibitory neurotransmitter in the mature CNS. During early development, however, GABA initially acts as an excitatory neurotransmitter before shifting to its inhibitory role in adulthood. The transformation is essential for proper neuronal maturation and the establishment of functional neural circuits (Hudson and Grau, 2022; Lam et al., 2023). GABA exerts its effects through three main receptor types: GABA_A_Rs, GABA_B_Rs and GABA_C_ receptors (GABA_C_Rs), which are expressed on both pre- and post-synaptic sites on neurons and glial cells (Bormann, 2000; Watanabe et al., 2002; Perovic et al., 2025). While GABA_B_Rs and GABA_C_Rs are mainly involved in inhibitory signaling, GABA_A_Rs can mediate inhibitory or excitatory effects depending on the chloride ionic (Cl^–^) gradient across the neuronal membrane (Bhagwani et al., 2022; Nascimento et al., 2024).

GABA_A_Rs are ionotropic, functioning as ligand-gated chloride ionic channels that enable rapid synaptic inhibition (Hevers and Lüddens, 1998; Miller and Smart, 2010). They are highly selective for chloride ions but also allow bicarbonate ions to pass, which can influence the pH within the postsynaptic neuron (Kaila and Voipio, 1987; Sigel and Steinmann, 2012). Usually, activation of GABA_A_Rs results in chloride ionic influx, hyperpolarizing the neuron and decreasing its likelihood of firing. However, in developing neurons or under certain pathological conditions, the chloride ionic gradient can shift, causing GABA_A_R activation to produce depolarizing currents. The depolarizing action plays a critical role during nervous system development and plasticity (Deidda et al., 2015; Ben-Ari, 2017).

GABA_A_Rs are pentamers made of combinations of subunits, α, β, γ, δ, ε, π, θ, and ρ, which shape their responses and functions (Bhagwani et al., 2022). When GABA binds, the receptor changes shape and opens chloride ionic channels, causing chloride ions to enter and affect postsynaptic potential. These receptors exist both at synapses (for quick, phasic inhibition) and outside synapses (responding to ambient GABA for sustained tonic inhibition) (Bhagwani et al., 2022; Hudson and Grau, 2022).

GABA_A_R activity is affected by a number of factors. Internal influences include gradients of chloride ions across the cell membrane. External drugs like benzodiazepines, barbiturates, and neurosteroids bind to allosteric sites on the receptor and modify its activity (Bhagwani et al., 2022). These compounds can boost or suppress receptor function, making GABA_A_Rs key targets for treating brain and mental health conditions (Farrant and Nusser, 2005; Belelli et al., 2009; Gupta et al., 2012). Studying how these receptors are modulated could be useful in the development of therapies to restore the inhibitory balance that is disrupted in many CNS disorders.

GABA_B_Rs differ from GABA_A_Rs in that they are metabotropic, belonging to the G protein-coupled receptor family. These receptors are highly conserved among vertebrates and primarily inhibit adenylyl cyclase activity, leading to reduced levels of cyclic adenosine monophosphate (Kaupmann et al., 1997). Activation of GABA_B_Rs also influences ion channels by suppressing voltage-gated calcium channels and promoting the opening of G protein-coupled inwardly rectifying potassium channels (Bettler et al., 2004). This results in neurons becoming hyperpolarized for longer durations, which diminishes their excitability (Knight and Bowery, 1996; Bettler et al., 2004).

GABA_B_Rs are situated on both pre-synaptic and post-synaptic membranes, playing essential roles in controlling neurotransmitter release and shaping post-synaptic responses. Drugs like baclofen and endogenous GABA activate these receptors, affecting synaptic plasticity and network activity. Because of their influence on neural circuits, GABA_B_Rs present promising targets for treating conditions such as epilepsy, muscle spasticity, and addiction (Malaguarnera et al., 2021; Witkin et al., 2024).

GABA_C_Rs, also called ρ-subunit GABA_A_Rs, form ligand-gated chloride ionic channels as well. They are pharmacologically distinct from classical GABA_A_Rs, showing insensitivity to bicuculline (a GABA_A_R antagonist) and baclofen (a GABA_B_Rs agonist). GABA_C_Rs have high GABA sensitivity, and minimal desensitization, and are mainly localized in the retina, where they are crucial for visual processing (Bormann, 2000; Watanabe et al., 2002; Bhagwani et al., 2022).

GABA_C_Rs are unique because they are made from homomeric ρ subunits (ρ1–ρ3) and mainly found in the CNS. It makes them important for precise control of inhibition in specific neural circuits (Malaguarnera et al., 2021; Witkin et al., 2024). Learning more about these receptors could improve our understanding of the diverse roles of GABA and reveal their potential for neuromodulation and targeted therapies for CNS diseases.

#### Ionic channel–related gamma-aminobutyric acid dysfunction

A central aspect of CNS dysfunction syndromes like CIDS involves disruptions in ion channel activity. Among these, voltage-gated calcium channels are particularly important because they influence a variety of neuronal processes. These channels allow calcium ions to enter neurons, a step that is critical for neurotransmitter release, muscle contraction, and gene regulation (Lanzetti and Di Biase, 2022). Within voltage-gated calcium channels, the α2δ3 subunits play especially vital roles in the early development of neural circuits, including the formation of GABAergic synapses. During this period, they help increase presynaptic GABA release, support synapse formation, and promote axonal growth in GABAergic interneurons. These mechanisms contribute to early network activity, which is essential for establishing and fine-tuning neuronal connectivity (Simms and Zamponi, 2014). Gaining insight into the function of α2δ3 subunits within ion channels is key to understanding the complex regulation of GABA signaling and might reveal new avenues for therapeutic targeting in CIDS.

*The role of α2δ3 subunits in calcium channels*

Voltage-gated calcium channels regulate many aspects of neuronal function, such as synaptic transmission. The auxiliary α2δ3 subunits are crucial for neuronal survival, synaptic recycling triggered by activity, and maintaining proper calcium currents (Schöpf et al., 2021). These subunits are especially important during early brain development, supporting the formation of GABAergic synapses and the growth of axons (Bikbaev et al., 2020). When α2δ3 levels increase in developing neurons, they enhance pre-synaptic GABA release, promoting synapse formation and facilitating neural network maturation (Bikbaev et al., 2020). By amplifying GABA signaling during these critical early stages, α2δ3 subunits help drive initial network activity, which is needed for creating and refining neuronal circuits (Bikbaev et al., 2020).

GABA_A_Rs, GABA_B_Rs, and GABA_C_Rs each play a key role in controlling neuron excitability, synaptic transmission, and network behavior. Their interaction with molecules like α2δ3 subunits of voltage-gated calcium channels shows the complexity of GABA signaling (Bikbaev et al., 2020). Understanding these processes helps us better grasp normal brain function and develop therapies to restore GABA activity in CNS disorders.

*Cation-chloride cotransporters*

The accumulation of ionic gradients is a central feature of toxic injury in white matter. Anoxia/ischemia induces cellular ionic deregulation caused by the failure of regulatory mechanisms such as ATPases and coupled ion exchangers (Stys et al., 1992).

Spinal reflex hyperactivity and reduced synaptic inhibition are commonly associated with spasticity after spinal cord injury. In adults, the activation of GABA_A_R and glycine receptors inhibits neurons as a result of low intracellular chloride ionic concentration, which is maintained by the KCC2 (Boulenguez et al., 2010).

The inhibitory function of GABA signaling is deeply influenced by the regulation of intracellular chloride ionic concentration ([Cl^–^]_i_), which is primarily governed by the KCC2 in the mature CNS (Ferrini et al., 2020; Huang et al., 2024b; Raveendran et al., 2024). Although GABA is widely recognized as an inhibitory neurotransmitter, its post-synaptic effects are contingent upon the precise regulation of intracellular chloride ionic concentration, maintained through the dynamic interaction between two cation-chloride cotransporters: KCC2 and NKCC1 (Na^+^-K^+^-2Cl^–^-cotransporter 1) (Ferrini et al., 2020; Talifu et al., 2022; Lam et al., 2023; Pressey et al., 2023; Cho et al., 2024). The balance between KCC2-mediated chloride ionic extrusion and NKCC1-mediated chloride ionic influx is critical for modulating neuronal excitability and synaptic plasticity, with intracellular chloride ionic concentration serving as a key determinant of GABA signaling efficacy (Ferrini et al., 2020; Huang et al., 2024b; Raveendran et al., 2024). Understanding the balance is crucial for elucidating the mechanisms underlying GABA dysfunction in CIDS and identifying potential therapeutic avenues for recovery.

During early neural development, the expression patterns of KCC2 and NKCC1 show clear temporal and spatial shifts. In immature neurons, high NKCC1 activity causes elevated intracellular chloride ionic concentration due to chloride ionic influx, often surpassing the capacity of KCC2 to extrude chloride ions. The imbalance leads to GABA responses that are depolarizing because, when GABA_A_Rs open, chloride ions flow out instead of in (Kaila et al., 2014; Campbell et al., 2015; Tillman and Zhang, 2019). As development proceeds, KCC2 expression gradually increases, typically beginning around embryonic day 13.5 in rodents. The rise coincides with the end of GABAergic neuronal precursor migration in the forebrain. In mature neurons, the dominant activity of KCC2 and the reduced function of NKCC1 maintain low intracellular chloride ionic concentration, thereby enabling GABA_A_R activation to produce hyperpolarizing chloride ionic influx, which sustains inhibitory signaling (Tillman and Zhang, 2019).

KCC2 is chiefly expressed in adult neurons of the CNS and is instrumental in extruding chloride ions, thereby supporting hyperpolarization and inhibition (Kaila et al., 2014; Hudson and Grau, 2022; Pressey et al., 2023). Beyond its well-known role in inhibitory neurotransmission, KCC2 has also been linked to modulating long-term potentiation at glutamatergic synapses, indicating its role in excitatory synaptic plasticity (Pressey et al., 2023). Reduced KCC2 activity has been associated with enhanced N-methyl-D-aspartate (NMDA) receptor potentiation, mediated via tropomyosin receptor kinase B signaling pathways, revealing a complex interaction between inhibitory and excitatory neurotransmitter systems (Ferrini et al., 2020).

The activity of KCC2 is finely tuned by phosphorylation at multiple sites, which dynamically influences its function during both development and synaptic plasticity, as well as in various pathological states (Virtanen et al., 2021; Raveendran et al., 2024). Notably, phosphorylation at serine 940 (S940) enhances surface stability, promoting chloride ionic extrusion, while dephosphorylation at threonine 906 (T906) and 1007 (T1007) reduces activity (Kahle et al., 2013; Tillman and Zhang, 2019; Lam et al., 2023). External signaling pathways, such as N-methyl-D-aspartate receptor activation, can activate protein phosphatase 1, which dephosphorylates S940 and diminishes KCC2 function, potentially shifting GABA responses from inhibitory to depolarizing (Lee et al., 2011a; Hartmann and Nothwang, 2022).

Furthermore, the with no lysine kinase (WNK)-Ste20-related proline/alanine-rich kinase (SPAK)/oxidative stress response 1 (OSR1) pathway plays a significant part in regulating KCC2 by phosphorylating T906 and T1007, which inhibits chloride ionic extrusion (Kahle et al., 2005; Rinehart et al., 2009; Alessi et al., 2014; Tang, 2020). Overactivation of this pathway can lead to impaired inhibition and is implicated in disorders such as epilepsy, neuropathic pain, and traumatic brain injury, emphasizing its potential as a therapeutic target (Kahle et al., 2005; Rinehart et al., 2009; Tang, 2020).

The dynamic interplay between KCC2 and NKCC1 is crucial for maintaining intracellular chloride ionic concentration and determining the polarity of GABA signaling. During development, the transition from depolarizing to hyperpolarizing GABA responses corresponds to the upregulation of KCC2 and the downregulation of NKCC1. In the adult CNS, KCC2 significantly influences inhibitory neurotransmission and synaptic plasticity, with its activity being finely tuned by phosphorylation and kinase signaling pathways (Lam et al., 2023; Pol et al., 2023).

A deeper understanding of the regulatory mechanisms governing KCC2 and NKCC1 will provide essential insights into their roles in both healthy and diseased states of the CNS. Exploring these interactions opens new avenues for therapeutic interventions aimed at restoring GABA inhibition in a variety of neurological disorders. Such a strategy has the potential to lead to enhanced treatment of conditions characterized by dysfunctional GABA signaling, ultimately improving outcomes for affected individuals.

### Immune dysfunction after central nervous system injury

The emerging recognition of the interplay between the peripheral immune system and the brain continues to shed light on complex neuroimmune interactions that are vital for maintaining brain health and understanding neurological diseases (Zhang et al., 2023).

CNS injury provokes an immediate activation of innate immune responses within the brain, often setting off a series of inflammatory processes. While these initial responses are meant to be protective, they sometimes evolve into systemic immunodepression, which increases vulnerability to secondary infections (Bouras et al., 2022). Damage to brain structures, such as meningeal contusion, axonal damage, or cerebrovascular disruption, triggers glial cell activation. These cells release a variety of pro- and anti-inflammatory mediators, which influence the local tissue environment (Colton, 2009).

The immune response also involves the recruitment of adaptive immune cells into the CNS (Zheng et al., 2025). B cells can produce autoantibodies after injury, contributing to autoimmune-like responses, while T cells release cytokines that support neuroprotection and help facilitate monocyte infiltration into the injured tissue (Ellwardt et al., 2016). Recent research highlights the protective and regenerative functions of T cells in neurological diseases, functions that are highly dependent on the specific subsets, especially CD4^+^ T cells (Evans et al., 2019; Zhang et al., 2023). Interestingly, self-reactive T cells have been linked to improved recovery, which may be mediated through their influence on myeloid cells (Salvador and Kipnis, 2022).

After CNS injury, the immune system shifts. Brain trauma causes local and systemic immune changes that lead to imbalances, immunodepression and infection risk. Damage releases molecules like damage-associated molecular patterns that activate immune cells, making monocytes pro-inflammatory (Jeffries and Tom, 2021; Duan et al., 2024).

Lymphocytes also change. T cells and regulatory T cells show altered activity, reducing immune control. Antibody production drops, weakening defenses. These peripheral changes trigger inflammation, which is worsened by microglia in the brain (Wang et al., 2024a; Zhang et al., 2025).

Microglia produce cytokines such as IL-1β, IL-12, TNF-α, C–C motif chemokine ligand 2 (CCL2), and C–X–C motif chemokine ligand 9, shaping inflammation (Loane and Kumar, 2016; Qiu et al., 2025). IL-1β and IL-6 attract immune cells to the injury, increasing inflammation (Nizamutdinov and Shapiro, 2017). Astrocytes respond to IL-1β, further amplifying immune signals and cytokines, which can destabilize balance and promote CIDS (Bird, 2017; Bouras et al., 2022; Sribnick et al., 2022; Dobson et al., 2024).

Reactive astrocytes release cytokines like TNF-α, IL-6, interferon β, CCL2, CCL5, and transforming growth factor β. While glial activation can support healing, it also boosts systemic inflammation and disrupts immune regulation (Eroglu and Barres, 2010; Liddelow and Barres, 2017; Sharma et al., 2019).

The immune response after CNS injury is complex. Early inflammation helps repair but can become imbalanced, causing chronic inflammation and immunodepression (Salvador and Kipnis, 2022). Understanding how glial activity and cytokines affect the balance is key to developing effective therapies for conditions such as CIDS.

Recent research shows that long-term inflammation plays a key role in symptoms linked to ongoing immune activation, often called “Brain FADE syndrome.” FADE syndrome refers to the combined endpoint of depression, fatigue and brain fog caused by chronic inflammation, which is named brain decline syndrome (Hanafy and Jovin, 2024). The condition is seen in many neurological disorders and includes cognitive impairment, asthenia and depression, among other symptoms, and is associated with persistent inflammation. Traditional psychiatric treatments often do not fully help, so new approaches that target both innate and adaptive immune responses are needed (Hanafy and Jovin, 2024). For instance, in post-traumatic stress disorder, ongoing inflammation is a critical factor and may result from injury (Jacquens et al., 2022). Using etanercept, which blocks TNF-α, has shown promising results for improving Brain FADE symptoms (Page, 2013).

Therefore, exploring the complex interactions between immune disruption following CNS injury and the emergence of CIDS is essential for designing effective treatments. Such therapies could restore immune balance and improve recovery trajectories.

### Neuroimmune interactions in central nervous system injury–associated immunodepression syndrome development

Although the brain was once regarded as an immune-privileged organ, recent insights have changed this view. It is now understood that the CNS maintains dynamic communication with the peripheral immune system through an elaborate network of neuroimmune pathways. Emerging evidence reveals structural and functional similarities between the CNS and secondary lymphoid organs, such as the presence of pseudo-germinal centers and resident immune cell populations, challenging the long-held idea of immune segregation within the brain. Conditions such as traumatic brain injury, stroke, and spinal cord injury involve chronic inflammation, which acts as a common pathological feature, characterized by systemic immunodepression and localized neuroinflammation that contribute to CIDS development (Parikh et al., 2020; Hanafy and Jovin, 2024).

Neuroimmune crosstalk relies on intricate networks of signaling pathways, with the autonomic nervous system serving as a key intermediary in CNS-immune system communication. Lymphoid tissue innervation by the sympathetic nervous system governs immune cell dynamics through norepinephrine release, a process that suppresses lymphocyte proliferation and promotes anti-inflammatory cytokine profiles (e.g., IL-10 induction) via β-adrenergic receptor signaling. In contrast, vagal-mediated parasympathetic signaling activates the cholinergic anti-inflammatory axis, constraining macrophage production of pro-inflammatory mediators (e.g., TNF-α, IL-1β) through α7 nicotinic acetylcholine receptor engagement (Duan et al., 2024).

Neuroendocrine signaling further integrates the stress response with immune regulation. Activation of the hypothalamic-pituitary-adrenal axis during injury leads to glucocorticoid secretion, which exerts immunosuppressive effects by inhibiting immune cell activation and cytokine biosynthesis. Concurrent alterations in immune cell trafficking occur as well, with pathological blood-brain barrier permeability enabling peripheral immune cell infiltration into the CNS. Additionally, CNS-derived inflammatory mediators (e.g., high mobility group box-1) propagate systemic immune responses via lymphatic and glymphatic pathways (Balakin et al., 2025).

Dysfunction within the GABA system represents a key pathological node linking CNS injury to systemic immunodepression. Post-injury loss of GABA inhibition disrupts both central and peripheral immune homeostasis. Within the CNS, GABA depletion promotes microglial and astrocytic hyperactivation, driving neuroinflammation and blood-brain barrier disruption. Systematically, brainstem-spinal GABAergic neurons regulate autonomic outflow, and injury-induced GABA depletion exacerbates sympathetic hyperactivity. Excessive catecholamine release (e.g., norepinephrine, epinephrine) induces lymphocyte apoptosis, defective antigen presentation, and cytokine imbalance, hallmarks of CIDS (Bhandage and Barragan, 2021; Wen et al., 2023).

Microglia link the local and systemic inflammation. Peripheral cytokines such as IL-6 and TNF-α increase microglial activation, worsening neuroinflammation (Wei et al., 2024). Gut microbes produce metabolites such as short-chain fatty acids, which influence microglia via the vagus nerve, highlighting the gut–brain link in immune regulation (Huang et al., 2023).

The cycle of immunodeppression and neuroinflammation continues. Breaking it requires therapies that target the communication between GABA signals and the immune response. The next sections examine how microglia mediate the exchange and their role in CIDS. Understanding these mechanisms is key to developing therapies that restore immune balance and reduce brain inflammation after CNS injury.

#### Roles of gamma-aminobutyric acid in immune regulation

GABA signaling functions as a crucial interface linking neural activity to immune responses. Beyond its role as the primary inhibitory neurotransmitter in the brain, GABA influences immune processes through multiple mechanisms. Its receptors are expressed on many immune cells, including T lymphocytes, macrophages, dendritic cells, and natural killer cells, allowing GABA to directly modulate cell activation, cytokine production, and effector functions (Bhandage and Barragan, 2021).

Especially within innate immunity, GABA plays a significant role. Monocytes and macrophages produce GABA (Stuckey et al., 2005) and respond to it by enhancing autophagy, promoting the maturation of phagosomes, and strengthening antimicrobial responses, particularly against intracellular bacteria. Notably, bacterial infections can reduce GABA levels within macrophages, both *in vivo* and *in vitro*; exogenously supplied GABA or GABAergic drugs have been shown to stimulate autophagy, assist phagosomal maturation, and improve antimicrobial activity against pathogens such as mycobacteria (Kim et al., 2018).

Neutrophils and dendritic cells also harbor GABA receptors. For instance, GABA_A_Rs and GABA_B_Rs on neutrophils facilitate chemotaxis via serine/threonine kinase B (Akt)-dependent pathways (Rane et al., 2005). Dendritic cells express GABA_A_Rs, GAD-65, and GABA transporter 4, and are capable of secreting GABA themselves (Kanatani et al., 2017). While GABA enhances their chemotactic responses and motility, it also appears to suppress their capacity to present antigens effectively (Fuks et al., 2012)). Emerging evidence suggests that mast cells may express GABA transporter 1, and that GABA transporter 2 influences IL-1β production in macrophages, indicating an extensive role for GABA signaling in innate immune regulation (Plum et al., 2020; Xia et al., 2021).

GABA affects not only innate but also adaptive immunity. T cells, which have neurotransmitter receptors, adjust their activity in response to signals like cytokines and neurotransmitters (Ganor and Levite, 2014). GABA can suppress T cell growth and lower pro-inflammatory cytokines by inhibiting pathways such as mitogen-activated protein kinase and nuclear factor kappa-B (Wu et al., 2017; Wang et al., 2018). In activated T cells, GABA transporter 1 is significantly expressed. Its activity has been linked to a reduction in CD4^+^ T cell proliferation, highlighting its role in T cell immunity (Wang et al., 2018).

GABA_A_R activation has been shown to inhibit T cell responses to antigens, both in laboratory settings and in living organisms. The effect is likely due to interference with the T cell receptor/CD3 signaling pathway and suppression of *IL-2* gene expression (Stuckey et al., 2005). Since subunits of GABA_A_Rs subunits are expressed on both CD4^+^ and CD8^+^ T cells, their activity influences T cell proliferation and function (Mendu et al., 2011). Agonists targeting GABA_B_Rs, such as baclofen, have demonstrated the ability to restore critical aspects of immune function in models of immunodepression, improving delayed hypersensitivity responses, increasing thymus size, and boosting thymocyte counts. These effects appear to occur through direct GABA_B_R activation on immune cells and by restoring neural regulation of immune responses (Tiurenkov et al., 2008).

GABA signaling may also suppress autoimmunity and reduce the activity of antigen-presenting cell activity, which is involved in inflammatory diseases including rheumatoid arthritis (Tian et al., 2011). Additionally, GABA and its related disorders have been implicated in pain and autoimmune conditions, such as stiff-body diseases like type 1 diabetes (Navarro-Ledesma et al., 2024). Type 1 diabetes, recognized as an autoimmune disorder, primarily involves immune responses against GAD-65 (Towns and Pietropaolo, 2011). The presence of anti-GAD antibodies is not limited to type 1 diabetes; similar immune responses are observed in various CNS disorders categorized as GAD-antibody spectrum diseases (Tsiortou et al., 2021). These observations highlight the potential for GABA-targeted strategies to treat immune-related conditions (Wu et al., 2017; Shan et al., 2023).

The effect of GABA on immunity depends on context. After brain injury, GABA levels drop, causing immune suppression, fewer lymphocytes, less antigen presentation, and shifts to anti-inflammatory cytokines like IL-10 and transforming growth factor β. Stimulating GABA_B_Rs with drugs like baclofen can help rebuild thymus structure, boost T cells, and improve immune responses (Tiurenkov et al., 2008). These mechanisms involve GABA receptors on immune cells and rebalancing the autonomic nervous system.

GABAergic neurons in the brainstem and spinal cord control sympathetic activity toward immune tissue. Injury reduces GABA, leading to overactive sympathetic signals that suppress lymphocytes and promote cell death via β-adrenergic pathways, while increasing anti-inflammatory cytokines. Reduced GABA also lowers vagal tone, weakening the anti-inflammatory pathway that uses α7 nicotinic acetylcholine receptor to suppress macrophages (Yao, 2022; Sudhakar, 2023).

GABA in the hypothalamus influences the hypothalamic-pituitary-adrenal axis. After injury, less GABA raises cortisol, which suppresses immune cells and cytokines through glucocorticoid receptors. The neuroendocrine disruption contributes to severe immunodepression in CIDS (Balakin et al., 2025).

GABA also controls the movement of immune cells across the barriers. If GABA signaling weakens, the blood–brain barrier becomes more permeable due to more matrix metalloproteinases, allowing immune cells into the brain. GABA affects chemokines that direct lymphocytes; for example, GABA can increase CCL21 in lymph nodes, helping dendritic cells move and present antigens (Liu et al., 2025).

GABA affects the gut–immune system. Gut bacteria such as Lactobacillus reuteri produce GABA, which affects the gut barrier and macrophages. Vagal signals carry gut GABA to the brain, affecting microglia. The gut-brain communication helps keep the immune balance (Liu et al., 2023, 2025).

Overall, GABA dysfunction drives neuroimmune problems in CIDS. Restoring GABA signaling could help fight immunodepression and neuroinflammation by normalizing nervous system activity, balancing the hypothalamic–pituitary–adrenal axis, and modulating immune cells. Next sections will explore how GABA affects microglia and neuroinflammation, revealing potential treatments for CIDS.

#### Association between gamma-aminobutyric acid and microglia

The CNS, once thought to be a site of immunological privilege, has been found to harbor immune-competent cells and to communicate with the peripheral nervous system. In the CNS, glial cells display immunological responses to pathological and physiological stimuli through pro- and anti-inflammatory cytokine and chemokine signaling, antigen presentation, and clearance of cellular debris via phagocytosis. Persistent neuroinflammation has been recognized as a major pathological component of virtually all neurodegenerative diseases and has been a focus of research into the pathology underlying psychiatric disorders. Accumulating evidence suggests that GABA activities are closely bound to immune processes and signals, and thus the GABA neurotransmitter system might represent an important therapeutic target in modulating neuroinflammation (Crowley et al., 2016).

Microglia, the resident immune cells in the brain, are directly affected by GABA through its receptors. Activation of microglial GABA_A_Rs promotes migration and superoxide release, while inhibition of GABA_B_Rs suppresses inflammatory cytokine secretion. The dual regulatory role positions the GABA system as a crucial modulator of neuroinflammation. For example, GABA binding to GABA_A_Rs on T cells suppresses their proliferation and diminishes the production of pro-inflammatory cytokines, including IL-6, IL-12, inducible nitric oxide synthase, IL-1β, and TNF-α (Bhandage and Barragan, 2021; Sparrow et al., 2021). Furthermore, GABA_A_R activation has been shown to inhibit TNF-α and IL-6 release from alveolar macrophages stimulated by lipopolysaccharide (Shan et al., 2023).

A recent study shows that lipopolysaccharide increases GABA uptake in microglia and changes GABA transporter 1 transport. It suggests inflammation affects GABA clearance (Bhandage et al., 2019). Also, GABA transporter 1 and Bestrophin-1 interactions are new pathways in brain inflammation. Activating GABA_A_Rs can protect against inflammation in models of multiple sclerosis (Tian et al., 2018).

Microglia are not merely passive recipients of GABA signaling; they actively participate in GABA metabolism and regulation within the brain. These cells possess the complete machinery for GABA synthesis, release, and uptake, including GABA-producing enzymes, GABA_A_Rs and GABA_B_Rs, and GABA transporters 1 and 3 (Lee et al., 2011b; Bhandage et al., 2019; Fattorini et al., 2020; Bhandage and Barragan, 2021). The capacity places microglia as active players in maintaining GABA dynamics, challenging earlier views that primarily associated GABA interactions with neurons and astrocytes (Fattorini et al., 2020).

#### Influence of microglia on gamma-aminobutyric acid signaling

Microglia, which are the innate immune cells within the CNS, play an essential part in regulating GABAergic signaling and shaping inhibitory neural circuits (Vukovic et al., 2012; Kreisel et al., 2019). These cells actively participate in synapse formation, maturation, and removal by directly interacting with synapses, engaging in phagocytosis, and secreting various signaling molecules. When microglial regulation is disturbed, it can lead to synaptic dysfunction and behavioral changes, emphasizing their importance in preserving neural stability (Andoh and Koyama, 2021; Lewis, 2021). A recent study has uncovered a specific subset of microglia that respond to GABA and selectively associate with inhibitory synapses during crucial stages of development (Favuzzi et al., 2021). Activation of GABA receptors in these microglia prompts a transcriptional response that fosters synapse remodeling, thereby strengthening inhibitory connections while sparing excitatory ones (Favuzzi et al., 2021).

In conditions such as traumatic brain injury, microglial activity can become maladaptive, resulting in loss of inhibitory synapses. When activated, microglia release brain-derived neurotrophic factor, which interacts with the tropomyosin receptor kinase B receptor on neurons. The binding triggers signaling pathways involving extracellular signal-regulated kinases 1 and 2 and glycogen synthase kinase 3β, leading to the phosphorylation and subsequent degradation of gephyrin, an essential scaffolding protein at inhibitory sites. The degradation of gephyrin impairs inhibitory synapses and disrupts normal neural circuits (Andoh and Koyama, 2023). Additionally, systemic inflammation prompted by lipopolysaccharide can stimulate microglia to engulf neuron bodies and strip away inhibitory pre-synaptic structures, which may increase neuronal synchrony and contribute to network hyperexcitability (Chen et al., 2014; Andoh and Koyama, 2023).

Astrocytes aid GABA signaling and neuroinflammation. They release GABA, which affects microglia responses, linking GABA issues to immune problems after traumatic brain injury (Lee et al., 2011b; Witkin et al., 2024). The GABA-glia interaction involves complex regulation, offering new treatment ideas for traumatic brain injury.

GABA and microglia interact in controlling inflammation, metabolism, and synaptic changes. These roles highlight the importance of the GABA system in linking neural and immunological functions, making it a potential therapeutic target for CNS conditions.

#### Gamma-aminobutyric acid regulation of the gut-brain axis and homeostasis of the gut

The gut-brain axis is crucial for immune regulation, with GABA playing a significant role in the complex signaling network through interactions with enteric neurons, enteroendocrine cells, and immune cells (Mazzoli et al., 2017). Disruptions in intestinal homeostasis and increased intestinal permeability are early immune-mediated events observed in autoimmune disorders, including experimental autoimmune encephalomyelitis, inflammatory bowel disease, and celiac disease (Suenaert et al., 2002; Nouri et al., 2014). GABA signaling may facilitate the restoration of intestinal barrier function and modulation of immune responses, presenting a promising therapeutic avenue for gut-related autoimmune disorders (Mazzoli et al., 2017).

## Immunodepression Mediated by the Gamma-Aminobutyric Acid System in Brain Disorders

Emerging evidence indicates that immune dysregulation is a key factor in the pathophysiology of traumatic brain injury, shock, and various CNS diseases, including neurodegenerative diseases, schizophrenia, type 1 diabetes, multiple sclerosis, and spinal cord injury. The GABA system not only modulates neural inhibition, but also plays a significant role in regulating the immune response, highlighting its critical involvement in maintaining both neural and immune homeostasis.

### Gamma-aminobutyric acid system and immunodepression after traumatic brain injury

Traumatic brain injury disrupts normal neurotransmission, especially GABA pathways, leading to immunodepression. Damage decreases GABA neurons, reducing inhibition, which worsens neuroinflammation and secondary injury (Crowley et al., 2016; Di Palma et al., 2023). GABA controls neuronal activity; when levels fall, inhibition weakens, allowing excess glutamate and excitotoxicity, key features of traumatic brain injury (Guerriero et al., 2015; Witkin et al., 2024). The imbalance may cause post-traumatic epilepsy, cognitive issues, and immune problems (Guerriero et al., 2015; Golub and Reddy, 2022; Witkin et al., 2024).

Post-traumatic brain injury immunodepression resembles aging-related immune decline. In Alzheimer’s disease, chronic suppression hampers the removal of amyloid-beta and disrupts cell metabolism, speeding up the disease (Weekman et al., 2014; Salminen et al., 2018). Similarly, after traumatic brain injury, waste clearance slows, increasing neuroinflammation and tissue damage, raising long-term risks for cognitive decline (Guerriero et al., 2015).

A comprehensive understanding of how GABA deficits relate to neuroinflammation and systemic immune suppression is essential for developing targeted treatments. Such insights can guide the design of interventions aimed at restoring GABA activity, reducing neuroinflammation, and improving recovery prospects for patients suffering from traumatic brain injury.

### Gamma-aminobutyric acid dysregulation and immune responses in stroke

Research into stroke pathophysiology increasingly emphasizes the importance of GABA in promoting neurological recovery, suggesting avenues for further exploration (Perovic et al., 2025). In individuals who have experienced a stroke, GABA concentrations tend to decrease markedly between 3 and 12 months post-injury, compared to healthy individuals (Blicher et al., 2015). The decline may result from the loss of GABAergic neurons (Saji et al., 1994) and diminished expression of enzymes vital for GABA synthesis, particularly GADs (Lin et al., 2010).

Efforts to regulate GABA transmission, especially through short-term administration of GABA agonists like muscimol and baclofen, as well as GABA-modulating drugs such as benzodiazepines, have shown promise in reducing ischemic brain damage (Green et al., 2000; Schwartz-Bloom and Sah, 2001). For example, early treatment with diazepam following stroke has demonstrated success in preventing seizures, common complications after stroke, without significantly affecting mortality rates (van Tuijl et al., 2021). However, some research indicates that using GABA receptor inverse agonists during the acute phase could worsen stroke outcomes (Clarkson et al., 2010), suggesting that the effects of GABA release are complex and context-dependent, they can be neuroprotective or neurotoxic depending on timing and dosage (Hamill et al., 1983; Raimondo et al., 2017).

The role of GABA in post-stroke immune problems is a key area of research. A better understanding could lead to therapies that target GABA to help both brain repair and immune function.

#### Immune dysregulation after ischemic stroke

Stroke, mainly ischemic stroke, is a major cause of death and disability. It occurs when arteries block blood flow to the brain, causing lasting damage. Both innate and adaptive immunity are activated during stroke (Wang et al., 2024c). Initially, inflammation worsens brain injury, but later it can aid healing. Damage-associated molecular patterns affect the blood–brain barrier, allowing immune cells in, causing swelling and more damage (Stanzione et al., 2022).

Lymphocytes are key players in the immune response following a stroke. Analyses of blood samples from patients with ischemic stroke have documented increases in B cells, regulatory B cells and CD8^+^ regulatory T cells from the time of hospital admission through 3 months afterward. Meanwhile, CD4^+^ regulatory T cells initially decrease before later rebounding. Importantly, the proportion of CD4^+^ regulatory T cells, CD8^+^ regulatory T cells, and double-negative T cells are strongly associated with the extent of brain infarction and neurological recovery. When combined with basic clinical data, these lymphocyte populations enhance the accuracy of predicting long-term outcomes. Notably, the percentage of CD4^+^ regulatory T cells shows a significant correlation with both immediate and extended neurological status, indicating their potential use as biomarkers for stroke prognosis and as therapeutic targets to modulate immune responses (Li et al., 2021). Activation of regulatory T cells appears to offer protective effects against neuronal damage (Stanzione et al., 2022).

Recent studies have shown that ILs play a dual role in ischemic stroke, facilitating the communication, activation, and regulation of immune cells, while also mediating the activation, proliferation, and differentiation of T and B cells during the inflammatory response. Crosstalk among various ILs within different populations of immune cells further affects the outcome of ischemic stroke. Pathophysiological changes following ischemic stroke, including ionic imbalances, neuroinflammation, and abnormal immune cell activation, can lead to neuronal death (Zhu et al., 2022).

#### Immune disruption in hemorrhagic stroke

Hemorrhagic stroke, or intracranial hemorrhage, causes high death and disability rates. It involves bleeding into brain tissue and surrounding areas, affecting both brain matter and meninges. Early signs are hard to distinguish from ischemic stroke (Caceres and Goldstein, 2012). A severe type, subarachnoid hemorrhage, occurs when blood rapidly enters the space between the brain and its membranes, often from aneurysm rupture (Boling and Groves, 2019). Subarachnoid hemorrhage mortality can reach 50%, especially with aneurysms (Penn et al., 2015). It accounts for about 5%–9% of all strokes annually (Macdonald and Schweizer, 2017; Neifert et al., 2021; Wang et al., 2022).

Neuroinflammation, primarily driven by lysed red blood cells releasing damage-associated molecular patterns such as heme, methemoglobin, and high mobility group box-1, underlies cerebral injury following subarachnoid hemorrhage. These damage-associated molecular patterns bind to Toll-like receptor 4, triggering inflammatory responses that lead to early brain injury and poor prognoses (Wang et al., 2022). Toll-like receptor 4 is crucial for neuronal damage and cognitive deficits in subarachnoid hemorrhage models and is predominantly expressed in microglia (Hanafy, 2013). As an integral component of the innate immune system, Toll-like receptors act as recognition receptors activated by damage-associated molecular patterns. Subarachnoid hemorrhage induces microglial migration into the affected brain region, leading to secondary neuronal injury that significantly contributes to cell death. Notably, microglial depletion using clodronate reduces the injury, highlighting Toll-like receptor 4 on microglia as a promising therapeutic target for subarachnoid hemorrhage (Islam et al., 2022).

### Gamma-aminobutyric acid dysfunction and immune dysregulation in neurodegenerative diseases

The GABA system is essential in the pathophysiology of neurodegenerative diseases such as Alzheimer’s disease and Parkinson’s disease. In the 5×FAD mouse model, which harbors five familial Alzheimer’s disease gene mutations, researchers observed significant reductions in gamma oscillation power during hippocampal sharp wave ripples. The decline correlates with impairments in cognitive function and sensory processing, underscoring the role of GABA signaling in maintaining neural network integrity (Barbour et al., 2024).

Gamma oscillations, controlled by GABA interneurons, influence microglia. When hippocampal gamma activity is triggered by stimuli, genes for microglial phagocytosis and migration are up, helping clear amyloid-beta in Alzheimer’s disease. Picrotoxin, a GABA_A_R blocker, stops the effect, highlighting the key role of GABA in amyloid removal (Barbour et al., 2024).

In Parkinson’s disease, glial cells show chronic inflammation, marked by IL-2, IL-6, and TNF-α, linked to neuron loss in the substantia nigra (Mogi et al., 1994, 1996). The inflammation contributes to disease development. Researchers also study inflammatory biomarkers for diagnosis, using cell and animal models (Chen et al., 2023).

Overall, the roles of GABA in neurodegeneration are dual: supporting cognition through gamma oscillations and helping microglia clear harmful proteins. When GABA signaling weakens in Alzheimer’s disease, cognition worsens, and inflammation and neuron loss increase. Targeting GABA could treat both problems.

### Gamma-aminobutyric acid and immune issues in schizophrenia

Schizophrenia involves less GABA in the brain, reducing inhibition and increasing dopamine activity, linked to psychosis. Less GABA in areas like the midbrain disrupts control, fueling inflammation and worsening symptoms (Purves-Tyson et al., 2021; Năstase et al., 2022). GABA deficits affect both brain function and immune responses (Guerriero et al., 2015; Purves-Tyson et al., 2021; Năstase et al., 2022).

Immune changes include higher IL-4, IL-13, monocyte chemoattractant protein-1, and lower IL-10. Patients often have more C–X–C motif chemokine ligand 8 and less paracetamol. Some treatments may partially normalize these factors (Arabska et al., 2022). These links show how GABA problems connect to immune dysregulation in schizophrenia.

### Gamma-aminobutyric acid dysregulation and autoimmune conditions

GABA, widely recognized as the primary inhibitory neurotransmitter in the CNS, also plays an important part in peripheral immune regulation. For instance, in type 1 diabetes, GABA has been shown to suppress effector T cell responses toward beta cell antigens and to inhibit T cell receptor-mediated cell cycle progression. Treatment with GABA has been observed to slow the progression of type 1 diabetes in NOD/SCID mice and to reduce proinflammatory T cell responses in type 1 diabetes-prone NOD mice, even after autoimmune activity is established (Tian et al., 2004). Such immunodepressive effects suggest that GABA may prolong the early, prodromal phase of type 1 diabetes, offering a potential strategy for managing autoimmune diseases through GABA modulation.

In the experimental autoimmune encephalomyelitis model, which simulates multiple sclerosis, immune cells can produce GABA and express functional GABA receptors. The indicates that GABA might serve paracrine or autocrine functions in immune regulation. Enhancing GABA activity in the context has shown promise in alleviating paralysis by dampening inflammation, highlighting the therapeutic potential of GABA-targeted interventions in multiple sclerosis (Bhat et al., 2010). Similarly, GABA administration has been found to lessen the severity of collagen-induced arthritis, supporting its protective role across several autoimmune disorders (Tian et al., 2011).

### Gamma-aminobutyric acid dysfunction and immune abnormalities in depression, epilepsy, and neuropathic pain

Disruptions in GABA and glycine signaling have been implicated in a variety of neurological and psychiatric conditions, including depression, epilepsy, and neuropathic pain (Schmidt and Thompson, 2016; Kim and Yoon, 2017; Krueger-Burg, 2025). Reduced GABA_A_R density has been documented in the temporal lobes of individuals with generalized anxiety disorder (Gross and Hen, 2004). Similarly, patients with panic disorder show decreased GABA_A_R numbers in regions such as the parietal, temporal, and frontal cortices, as well as in the hippocampus and precuneus (Fatemi and Folsom, 2015). An animal study further supports these findings; infusions of GABA or GABA_A_R agonists into the amygdala reduce anxiety behaviors, while antagonists produce anxiogenic effects (Nuss, 2015).

The central role of GABA in anxiety regulation is well established, making the neurotransmitter system a primary target for benzodiazepines and related medications that effectively treat anxiety disorders (Lydiard, 2003). Current antidepressants not only enhance monoaminergic transmission but also strengthen GABA transmission, which is often diminished in anxiety disorders (Kalueff et al., 2007). Addressing GABA dysfunction may therefore represent a critical strategy for developing more effective treatments for these interconnected conditions.

### Gamma-aminobutyric acid function and immune regulation by age and sex

Age and sex affect GABA signals in the brain. With age, GABA-related gene expression drops, weakening inhibition and reducing plasticity and recovery after injury (Loerch et al., 2008; Lehmann et al., 2012). Conversely, aging also increases GABA inhibition, especially via GABA_B_Rs (Opie et al., 2018). Sex differences exist in GABA receptor subunit expression during the aging process, showing gender-related variability (Ethiraj et al., 2021). GAD, the enzyme for GABA production, remains mostly stability, but in older women, GAD-65 can decrease (Ethiraj et al., 2021). More research is needed to understand how these changes influence CNS diseases and inhibition types.

## Gamma-Aminobutyric Acid System and Spinal Cord Injury

Spinal cord injury disrupts the excitatory-inhibitory balance, leading to maladaptive plasticity within spinal sympathetic reflex circuits (SSRCs) and promoting systemic immunodepression. Following spinal cord injury, aberrant sprouting of excitatory interneurons increases the excitability of spinal sympathetic reflex circuits, causing heightened sympathetic output and sympathetic hyperreflexia (Brennan et al., 2024; Gillespie et al., 2024). The hyperreflexive response correlates with immune depression observed in both human patients and animal models of spinal cord injury (Zhang et al., 2013; Brennan et al., 2021, 2024). Clinically significant, therapeutic approaches to sympathetic hyperreflexia remain sparse due to the incomplete understanding of the mechanisms driving GABA inhibition loss and maladaptive plasticity in spinal sympathetic reflex circuits following spinal cord injury.

### Role of the gamma-aminobutyric acid system in the spinal sympathetic reflex circuits

The GABA system, including GABAergic interneurons, GABA receptors, isoforms of GADs (GAD-65 and GAD-67), the vesicular GABA transporter, and the KCC2, is crucial for maintaining integrity and functionality of spinal sympathetic reflex circuits. Interneurons located in the intermediolateral region, adjacent to sympathetic preganglionic neurons, precisely modulate inputs to these neurons, ensuring a delicate excitation-inhibition balance influences within the spinal cord (Sivilotti and Woolf, 1994; Talifu et al., 2022; Brennan et al., 2024).

Proper function of spinal sympathetic reflex circuits depends on coordinated GABA components. In the dorsal horn, GABA receptors control tonic inhibition of sensory inputs, balancing excitation and inhibition (Xu et al., 2023). Descending brain signals regulate both activity of spinal sympathetic reflex circuits and spinal GABA circuits. Disruptions here may impair GABA enzyme and receptor expression, affecting reflex responses (Russ et al., 2013; Mazzone et al., 2021).

Under normal conditions, reflex activation of sympathetic preganglionic neurons is tightly regulated through inputs from both the supraspinal centers and the spinal interneurons, ensuring sympathetic tone remains balanced. The ensures sympathetic tone homeostasis through integrated molecular and ionic mechanisms (e.g., KCC2/NKCC1 phosphorylation sites, glutamate-GABA cycle), and it serves as the foundational scaffold upon which signaling cascades like the WNK-SPAK/OSR1 pathway. Maintaining optimal function of spinal sympathetic reflex circuits relies on the intricate coordination of these structural relationships and key molecular players.

#### Critical projection and primary afferent fibers

From an anatomical perspective, the preservation of the structural and function of the spinal sympathetic reflex circuits heavily depends on supraspinal neurons originating from the brainstem, as well as peripheral afferent fibers that respond to visceral and somatic stimuli (Noble et al., 2022; Brennan et al., 2024). These neural pathways serve to transmit and modulate sensory and autonomic signals within the spinal cord, emphasizing the importance of intact GABA signaling for maintaining homeostasis in spinal sympathetic pathways. Exploring these mechanisms could open avenues for novel therapeutic strategies aimed at correcting dysregulation, especially following spinal cord injury.

#### Efferent fibers from the rostral ventrolateral medulla

Neurons in the rostral ventrolateral medulla project fibers that innervate sympathetic preganglionic neurons in the spinal cord, playing a vital role in controlling both basal and reflex sympathetic activity. Located bilaterally in the brainstem, the rostral ventrolateral medulla provides GABAergic input to sympathetic preganglionic neurons on both sides of the spinal cord. The inhibitory influence, mainly modulated by arterial baroreceptor pathways, affects splanchnic sympathetic nerve activity through GABA_A_Rs (Kulkarni et al., 2022; Moss et al., 2024; Saraswathy et al., 2024).

Within the rostral ventrolateral medulla, GABA inhibition facilitates bidirectional control of excitatory drives to sympathetic targets, including those involved in immune regulation (Schreihofer and Guyenet, 2002). A notable proportion of descending fibers of rostral ventrolateral medulla are GABAergic, with many co-expressing enkephalin. Optogenetic stimulation of these dual GABAergic/enkephalinergic neurons can induce dorsal root potentials, demonstrating their presynaptic influence on primary afferent fibers (Zhang et al., 2015; Comitato and Bardoni, 2021). The modulation illustrates the critical role of GABA signaling in integrating autonomic and sensory inputs within the spinal cord.

#### Primary afferent fibers and sensory input

Primary afferent fibers, such as Aδ and C fibers, carry signals from tissues to the spinal cord. They connect in the dorsal horn, where ion channels and receptors control glutamate and neuropeptide release, activating second neurons (Comitato and Bardoni, 2021).

In lamina I–III, about one-third of neurons are GABA interneurons. They modulate primary afferent fiber signals and sensory input (Heinke et al., 2004; Meisner et al., 2010). When primary afferent fibers fire, glutamate activates GABA interneurons, which release GABA. GABA binds to presynaptic GABA_A_Rs, regulating sensory signals. The process shows how presynaptic GABA_A_Rs work to shape dorsal horn sensory processing (Comitato and Bardoni, 2021; Bryson et al., 2025).

#### Key interneurons and sympathetic preganglionic neurons

On a cellular and anatomical level, primary afferent fibers do not directly connect with sympathetic preganglionic neurons. Instead, sensory afferents influence sympathetic preganglionic neurons indirectly through interactions with spinal interneurons, which serve as crucial intermediaries within the spinal sympathetic reflex circuits (Balik and Šulla, 2022). The pathway underscores the importance of interneurons in linking sensory inputs to autonomic outputs, facilitating coordinated responses necessary for autonomic homeostasis.

These interneurons can act either to excite or inhibit the activity of sympathetic preganglionic neurons. The adaptability and plasticity of these interneurons have been a focus of ongoing research in the CNS (Wulf and Tom, 2023). In the spinal cord, three main types have been identified: GABAergic interneurons, those co-releasing GABA and glycine, and glycinergic interneurons. Collectively, they fulfill diverse roles from modulating sensory signals, and influencing motor functions, to regulating autonomic responses (Shimizu-Okabe et al., 2022; Brennan et al., 2024; Bryson et al., 2025).

GABAergic interneurons are primarily situated within lamina I and II of the dorsal horn, with some presence in the central spinal gray matter and fewer in the ventral horn. Interneurons that co-release GABA and glycine are mostly found in laminae III to VI, whereas glycinergic interneurons concentrate in laminae VII to IX (Shimizu-Okabe et al., 2022). Together, these inhibitory interneurons form a primary component of the inhibitory system of spinal cord, working alongside glutamatergic excitatory interneurons to maintain a balance between excitation and inhibition, known as the excitatory-inhibitory balance (Meisner et al., 2010; Takazawa et al., 2017; Brennan et al., 2024; Saraswathy et al., 2024).

Sympathetic preganglionic neurons are in the thoracolumbar spinal cord (T_1_–L_2_). They process inputs from brain centers and local interneurons, controlling postganglionic neurons that regulate organ and immune functions (Wulf and Tom, 2023).

Normally, reflexes activate sympathetic preganglionic neurons via brainstem and spinal interneurons, maintaining balanced sympathetic activity. Understanding these circuits may help develop therapies for spinal cord injury recovery (Noble et al., 2022; Brennan et al., 2024).

### Impact of spinal cord injury on the gamma-aminobutyric acid system and spinal sympathetic reflex circuits

Spinal cord injury causes notable disturbances within the GABAergic system, leading to dysfunction in the spinal sympathetic reflex circuits. Normally, the spinal sympathetic reflex circuits maintain a critical equilibrium between excitatory and inhibitory signals, essential for regulating sympathetic activity. After spinal cord injury, the plasticity of synapses within the circuit is often impaired, resulting in heightened excitability and reduced inhibitory control. Such imbalance can provoke excessive sympathetic reflex responses, such as hyperreflexia, and contribute to systemic immunodepression, as extensively documented in research literature (Mironets et al., 2018, 2020; Brennan et al., 2024).

The disruption of the GABAergic system is a core element underlying heightened excitatory activity within the spinal sympathetic reflex circuits. The imbalance worsens the abnormal functioning of the circuit and plays a pivotal role in the development of spinal cord injury–associated immunodepression syndrome. Several key alterations related to GABA following spinal cord injury have been identified. These include the loss of GABAergic interneurons, a decline in GABA levels, decreased expression of enzymes responsible for GABA synthesis such as GAD-65 and GAD-67, downregulation of the KCC2, reduced surface expression of GABA receptor subunits, and increased GABA reuptake mediated by vesicular GABA transporter activity (Bhagwani et al., 2022; Hudson and Grau, 2022; Li et al., 2022). Notably, research indicates that GAD-65 and GAD-67 expression in the dorsal horn diminishes approximately one month after contusive spinal cord injury, accompanied by microglial activation that further suppresses GABA tone (Bhagwani et al., 2022; Li et al., 2022).

#### Abnormal sprouting of primary afferent fibers after spinal cord injury

Following spinal cord injury, an abnormal growth of primary afferent fibers becomes apparent below the injury site. The sprouting is notably marked by an increase in calcitonin gene-related peptide (CGRP) positive fibers within the dorsal horn and in laminae VII and X surrounding the central canal (Mironets et al., 2018, 2020). The unusual outgrowth of pelvic visceral C-fibers facilitates the transmission of damaging visceral sensory inputs to disinhibited thoracic spinal preganglionic neurons located higher up in the spinal cord (Michael et al., 2019; Brennan et al., 2024). Post-injury, neurons can undergo genetic changes that modify their molecular expression and functions, a process referred to as a “phenotypic shift” (Michael et al., 2019).

The circuits involved in spinal sensory processing are notably affected during spinal cord injury. Nociceptive neurons, including Aδ and C fibers marked by calcitonin gene-related peptide, substance P, and tropomyosin receptor kinase A (TrkA), often experience disrupted signaling. Meanwhile, non-nociceptive Aβ fibers may begin to express calcitonin gene-related peptide and substance P, leading to alterations in their electrical behavior and neurochemical properties, diverging from their typical profiles (Comitato and Bardoni, 2021). As a consequence, inhibitory GABAergic interneurons in the superficial dorsal horn, which normally receive input from Aδ and C fibers, display decreased inhibitory control after injury. The reduction allows normally innocuous Aβ fibers to activate nociceptive-specific projection neurons and previously inactive low-threshold C-fiber circuits, resulting in heightened pain perception (Meisner et al., 2010).

Structural changes, such as calcitonin gene–related peptide fiber sprouting and primary afferent fiber phenotype shifts, greatly affect the activation of spinal sympathetic reflex circuits. These contribute to sympathetic overactivity and spinal cord injury–associated immunodepression syndrome (Mantyh et al., 2011; Mironets et al., 2018; Michael et al., 2019).

Elastic remodeling, involving plasticity in propriospinal fibers in the dorsal gray commissure and dorsal horn, enhances innervation between segments (Hou et al., 2008; Ueno et al., 2016; Michael et al., 2019). Studying these changes may lead to new treatments to restore normal sympathetic function.

#### Consequences of gamma-aminobutyric acid system loss after spinal cord injury

The reduction in GABAergic interneurons and the consequent decline in GABA concentrations following spinal cord injury substantially impair the integrity of spinal circuit. These changes are closely linked to the emergence of neuropathic pain, spasticity, and the progression of spinal cord injury–associated immunodepression syndrome (Balik and Šulla, 2022). Disruption of descending inhibitory signals leaves a greater reliance on interneurons within the intermediolateral region, which serve as crucial inputs to sympathetic preganglionic neurons (Tang, 2020; Wulf and Tom, 2023).

Following spinal cord injury, the suppression of inhibitory control from higher brain centers over sympathetic preganglionic neurons below the injury site often triggers compensatory changes within the interneuron circuits involved in sympathetic hyperreflexia (Ueno et al., 2016; Mironets et al., 2020). Among these interneurons, GABAergic cells, particularly commissural interneurons that project across the midline, appear to be more vulnerable to injury than glycinergic neurons. The increased susceptibility may relate to their specific anatomical distribution within the spinal cord (Mazzone et al., 2021).

After spinal cord injury, long-range propriospinal interneurons become more plastic, especially through the sprouting of nociceptive fibers. These interneurons connect across multiple segments, causing increased activation of sympathetic preganglionic neurons and related interneurons in the intermediolateral, which worsens sympathetic hyperreflexia (Hou et al., 2008; Ueno et al., 2016; Mironets et al., 2018).

Spinal cord injury also alters GABA interneurons, forming new synapses and reducing GABAergic cells. Levels of GAD-65 and GAD-67 decrease, while GABA reuptake increases via vesicular GABA transporters, lowering GABA in the synapse and weakening inhibition (Tai et al., 1997; Mazzone et al., 2021).

Reduced GABA inhibition causes glutamatergic interneurons to become more active, forming more connections with sympathetic preganglionic neurons in the spinal sympathetic reflex circuits (Ueno et al., 2016; Brennan et al., 2024). It raises circuit excitability, leading to hyperreflexia and possible immune issues (Michael et al., 2019; Mironets et al., 2020; O’Reilly and Tom, 2020; Brennan et al., 2024). Studying these changes may help develop therapies to restore GABA balance and improve spinal cord injury recovery.

#### Chloride ionic homeostasis disruption after spinal cord injury

Proper chloride ionic levels are crucial for neuron function. Transporters such as KCC2 and NKCC1 regulate intracellular chloride ions, affecting GABA signaling. Injury often reduces KCC2, shifting the GABA equilibrium potential (EGABA), making GABA currents more excitatory (Cheung et al., 2023; Capilla-López et al., 2024).

Lower KCC2 causes chloride ionic buildup inside cells, weakening inhibition and making GABA depolarizing, especially within 24 hours post-injury (Meisner et al., 2010). Chloride ionic levels remain high for days, impairing inhibition and promoting issues like spasticity and spinal cord injury–associated immunodepression syndrome (Boulenguez and Vinay, 2009; Huang et al., 2016; Bilchak et al., 2021a; Hudson and Grau, 2022; Brennan et al., 2024). Restoring KCC2 after spinal cord injury can improve motor function, suggesting it is a therapeutic target (Chen et al., 2018; Bilchak et al., 2021b; Mazzone et al., 2021).

#### Paradoxical role of gamma-aminobutyric acid A receptors after spinal cord injury

The expression and activity of GABA_A_Rs are not static; they change dynamically in response to injury and inflammatory signals. Laboratory studies have shown that pro-inflammatory cytokines like TNF-α can cause internalization of postsynaptic GABA_A_Rs (Stellwagen et al., 2005). However, animal models indicate that TNF-α and IL-1β may promote the movement of GABA_A_Rs to the cell membrane, increasing their surface presence and availability (Serantes et al., 2006; Stück et al., 2012).

Even when GABA_A_R levels rise, their ability to inhibit neurons drops in inflamed environments. It can flip the role of GABA from inhibitory to excitatory, increasing the firing of sympathetic preganglionic neurons and motor neurons (Huang and Grau, 2018; Bilchak et al., 2021b; Talifu et al., 2022).

These changes show a complex link between neuroinflammation and nerve activity after spinal cord injury. Treatments must address these paradoxes to restore normal function and improve outcomes.

#### Causes of gamma-aminobutyric acid problems after spinal cord injury

GABA helps keep neural activity balanced, but injury disrupts this. Levels often fall after spinal cord injury, linked to nerve damage, axon loss, cell death, and dendrite changes (Witkin et al., 2024). Causes include glutamate excitotoxicity, cycle disruption of glutamate-GABA, and glial activation (Balik and Šulla, 2022; Bhagwani et al., 2022; Brennan et al., 2024).

Glutamate excitotoxicity plays a central role in trauma-induced neural damage, driven by disruptions in inhibitory and excitatory signaling pathways. Trauma alters GABA signals, boosting glutamate activity and upsetting balance (Guerriero et al., 2015). Excess glutamate causes overactivation of receptors, leading to excitotoxicity and secondary damage. High glutamate increases hyperexcitability, raising risks like epilepsy, pain, and spasticity (Guerriero et al., 2015; Golub and Reddy, 2022). Early use of GABA-like drugs, such as gabapentin, can help prevent overexcitation and seizures if given soon after injury (Prince et al., 2016). Ongoing research aims to find drugs that restore GABA function and block excitotoxicity (Witkin et al., 2022, 2024). Glutamate toxicity involves calcium imbalance, vascular issues, ionic disturbances, and damaging reactive oxygen species, worsened by inflammation (Bhagwani et al., 2022). These factors lead to neuron loss, decreased GAD enzyme, increased GABA reuptake, and receptor overactivation. Strategies that boost GABA and reduce excitotoxicity could protect neurons, reduce pain, and lessen spasticity (Bhagwani et al., 2022; Li et al., 2022; Perovic et al., 2025).

Alterations in neurotrophic factors and disruptions in cellular cycles play a significant role in the pathophysiology following spinal cord injury. After spinal cord injury, glial cells stay active longer, releasing neurotrophins and cytokines that disrupt the glutamate-GABA-glutamine cycle. The ongoing inflammation causes maladaptive plasticity, leading to pain and autonomic dysreflexia (O’Reilly and Tom, 2020). Changes in neurotrophin levels affect synapse strength and neuron survival, complicating recovery. Targeting these factors may help repair nerves and improve outcomes. Understanding these mechanisms can also guide new treatments that address excitotoxicity and inflammation, aiming to boost GABA function after spinal cord injury.

Neurotrophic factors exert a significant influence on neuronal plasticity and reorganization processes following CNS injury, contributing to pain and dysreflexia. CNS injury often causes nerve reorganization in sensory and autonomic pathways, contributing to pain and dysreflexia (Cameron et al., 2006; Mironets et al., 2018, 2020). Increased nerve growth factor and brain-derived neurotrophic factor drives the plasticity (O’Reilly and Tom, 2020). After injury, TNF-α promotes microglia and macrophages to produce brain-derived neurotrophic factor, while also stimulating astrocytes to release nerve growth factor and brain-derived neurotrophic factor (Schulte-Herbrüggen et al., 2005; Kuno et al., 2006). Brain-derived neurotrophic factor influences pain sensitivity and central neuroinflammation (Xiong et al., 2024).

High nerve growth factor levels bind to TrkA on calcitonin gene-related peptide fibers, encouraging sprouting (Hou et al., 2008; Mantyh et al., 2011; Wulf and Tom, 2023). The structural change promotes neuropathic pain and dysreflexia (Cameron et al., 2006; Mironets et al., 2018, 2020). Blocking nerve growth factor-TrkA reduces calcitonin gene-related peptide activity, highlighting the role of TNF-α pathways in nerve sprouting (Mironets et al., 2018; O’Reilly and Tom, 2020).

Brain-derived neurotrophic factor from microglia also decreases KCC2 by acting on tropomyosin receptor kinase B receptors, reducing *KCC2* gene expression and chloride ionic extrusion after spinal cord injury (Huang et al., 2017; Bhagwani et al., 2022; Li et al., 2022). It impairs GABA inhibition, causing hyperexcitability and spinal cord injury–associated immunodepression syndrome (Gwak et al., 2017; Bhagwani et al., 2022). Restoring KCC2 regulation is key to re-establishing inhibition and controlling secondary damage. These factors show how injury triggers complex pathways that affect GABA and nerve function, emphasizing the need for new treatments.

Disruptions to the glutamate-GABA-glutamine cycle following spinal cord injury significantly impair inhibitory and excitatory balance within the central nervous system. In healthy states, astrocytes convert glutamate and GABA into glutamine via glutamine synthetase. Neurons use this to produce glutamate or GABA, maintaining balance (Schousboe et al., 2013). After spinal cord injury, inflammation disrupts it. Cytokines like TNF-α lower glutamine synthetase in astrocytes (Zou et al., 2010). GAD-65 production in astrocytes and microglia also drops, reducing GABA synthesis, as shown by less GABA staining in injured spinal cords (Gwak et al., 2008).

IL-10 and activated microglia increase vesicular GABA transporter, which lowers extracellular GABA, weakening inhibition (Bhagwani A, 2022). Astrocytes reuptake GABA, boosting glutamine and glutamate, which raises neuron excitability and causes maladaptive plasticity. These disturbances impair GABA systems, linked to neuropathic pain, spasticity, and spinal cord injury–associated immunodepression syndrome. Targeted therapies to restore balance could aid neural recovery after injury (Gwak et al., 2008; Zou et al., 2010; Brennan et al., 2024).

## Gamma-Aminobutyric Acid–based Treatments for Central Nervous System Injury–associated Immunodepression Syndrome

Targeting GABA pathways may help repair nerves and immune functions after CNS injury. New drugs, gene therapy, and stem cell methods aim to restore GABA activity, potentially reducing pain and spasticity (Bhagwani et al., 2022; Witkin et al., 2022, 2024).

### Focusing on potassium-chloride-cotransporter 2 for therapy

Hyperreflexia often follows CNS damage, linked to low KCC2 (Boulenguez et al., 2010; Huang et al., 2024b). The transporter keeps chloride ionic levels balanced, vital for the inhibitory role of GABA (Tang, 2020; Lam et al., 2023; McMoneagle et al., 2024; Nascimento et al., 2024, 2025). Boosting KCC2 could improve recovery and restore spinal neural balance (Chen et al., 2018; Bilchak et al., 2021a; Li et al., 2022; Talifu et al., 2022; Huang et al., 2024b).

Pharmacological agents that enhance KCC2 activity hold promise for reducing spasticity and hyperreflexia. Drugs that activate KCC2 could reduce spasticity and hyperreflexia (Ferrini et al., 2017, 2020; Hudson and Grau, 2022; Nascimento et al., 2024). Some compounds, called chloride ionic load protectors (CLPs), increase chloride ionic removal in neurons with low transporter activity (Gagnon et al., 2013; Ferrini et al., 2017; Chen et al., 2018). CLP290, shown in spinal cord injury models, restored walking in paralyzed mice and prevented KCC2 decreases in the dorsal horn of spinal cord (Chen et al., 2018; Lam et al., 2023). Similar compounds like CLP257 reduced spasticity in chronic spinal cord injury (Tang, 2020), offering a drug alternative or supplement to physical therapy (Beverungen et al., 2020; Bilchak et al., 2021a). These therapies could help patients unable to exercise.

Gene therapy targeting kcc2 offers a promising strategy for restoring chloride ionic homeostasis after spinal cord injury. Gene therapy also helps restore chloride ionic balance. Injecting the kcc2 gene into the spinal cord normalized chloride ions in neurons and dorsal root ganglion cells, easing neuropathic pain long-term (Li et al., 2016). The approach directly targets chloride ionic imbalance after spinal cord injury (Beverungen et al., 2020; Bilchak et al., 2021a).

Targeting the phosphorylation status of KCC2 presents a novel therapeutic avenue for modulating GABA signaling after spinal cord injury. How KCC2 phosphorylation affects its activity offers new therapy options for GABA signaling (McMoneagle et al., 2024). Protein kinase C (PKC) adds a phosphate to S940, stabilizing KCC2 on the cell surface and boosting its function (Lee et al., 2011a; Pressey et al., 2023). When protein phosphatase 1 removes the phosphate, KCC2 is internalized, reducing its activity (Pressey et al., 2023). Understanding these processes could lead to targeted treatments for spinal cord injury issues and new drug targets.

Focusing on KCC2 shows promise for recovery strategies. Increasing KCC2 activity via drugs or gene therapy has reduced spasticity and improved function in preclinical models (Bilchak et al., 2021b). Restoring chloride ionic balance and GABA inhibition may help patients unable to do exercise-based therapy, aiding recovery efforts.

### Role of activity-based training in recovery

CNS injuries cause lasting problems that lower quality of life. While many drugs are tested, exercise remains one of the best ways to help. Physical activity improves overall health by stimulating neural circuits, promoting dendrite growth, strengthening synapses, balancing neurotransmitters, and increasing neurotrophic factors. Repetitive, task-specific training is standard in clinics for functional recovery (Harkema et al., 2012).

Activity-based therapy supports neural recovery and reduces secondary issues. It uses high-frequency muscle and nerve activation through regular, moderate-to-high intensity exercise. Studying how activity causes brain plasticity in animals helps improve rehabilitative techniques and develop drugs for clinical use (Jervis-Rademeyer et al., 2024).

#### Using activity-based training to reduce spasticity by restoring gamma-aminobutyric acid

Exercise benefits many organs and raises brain-derived neurotrophic factor, which supports neurons (Reddy et al., 2023). Unlike some drugs, activity training is safe and helps restore GABA function after CNS injury. Studies show activity reduces spasticity by increasing molecules involved in GABA signaling, brain-derived neurotrophic factor, GABA, GAD-65 or GAD-67, and KCC2 (Beverungen et al., 2020; Li et al., 2020b; Bilchak et al., 2021a; Wang et al., 2024b). These molecules improve inhibition in the spinal cord, helping recovery and lowering spasticity in animal models (Beverungen et al., 2020; Li et al., 2020b, 2022).

Exercise increases brain-derived neurotrophic factor, which boosts KCC2 through the brain-derived neurotrophic factor-tropomyosin receptor kinase B pathway in injured neurons (Tashiro et al., 2015; Ma et al., 2021; Talifu et al., 2022). It also activates cyclic adenosine monophosphate response element-binding protein, especially its phosphorylated form, which helps regulate GAD-65 or GAD-67 gene expression via tropomyosin receptor kinase B signaling. The result is more GABA production and stronger inhibition (Bilchak et al., 2021a; Li et al., 2022). By restoring the natural inhibition, exercise may offer a safer alternative to drugs, reducing side effects and improving life quality in cases of spasticity (Tashiro et al., 2015; Ma et al., 2021; Talifu et al., 2022; Saraswathy et al., 2024).

Activity-based therapy with structured exercise reduces spasticity and aids recovery in CNS injury patients. It increases key GABA-related molecules like KCC2, GAD-65 or GAD-67, and brain-derived neurotrophic factor, strengthening inhibitory pathways important for rehabilitation (Beverungen et al., 2020; Li et al., 2022). Despite these benefits, widespread use is limited by cost and access issues. Many patients also face conditions that delay participation, highlighting the need for alternative treatments that mimic exercise effects.

Beyond symptom relief, activity-based training supports neural repair by promoting plasticity. It helps manage symptoms like spasticity and fosters long-term brain and spinal cord changes that improve function.

#### Using activity-based training to promote neurotransmitter plasticity

The nervous system can change throughout life, adapting to environmental and internal signals (Bertuzzi et al., 2018). One key form of plasticity is neurotransmitter switching, where neurons change the main transmitter, they produce (Spitzer, 2012; Li and Spitzer, 2020; Bertels et al., 2022; Godavarthi et al., 2024; Li et al., 2024; Pratelli et al., 2024). It can affect behavior and disease; for example, more dopamine helps Parkinson’s disease, while adjusting serotonin may relieve depression. Exercise may influence these changes, even if not fully recognized (Li et al., 2020a).

Research shows that glutamate and GABA neurons, sometimes co-expressing both transmitters, can form synapses with sympathetic preganglionic neurons (Llewellyn-Smith et al., 1997). Similar phenomena are seen in Parkinson’s and Huntington’s diseases (Ncube et al., 2022). Different stimulation patterns can cause neurons to switch neurotransmitters, reorganizing networks (Spitzer, 2015; Godavarthi et al., 2024; Li et al., 2024; Pratelli et al., 2024). Receptor changes often match these shifts, improving synapse function (Spitzer, 2012).

Pre-injury exercise increases GABA neurons in the spinal cord, making them more resistant to damage (Kiss Bimbova et al., 2023). It also boosts GABA function in the medulla that projects to spinal neurons (Fyk-Kolodziej et al., 2021; Kulkarni et al., 2022). Prolonged activity can cause lasting gene changes and neuron type switches, affecting network behavior (Bertuzzi et al., 2018; Li et al., 2020a; Bertels et al., 2022). For example, a week of running can turn midbrain neurons into GABAergic cells, improving motor learning (Li et al., 2020a).

Studies show sports rehabilitation can counteract GABA system damage from spinal cord injury (Bilchak et al., 2021b; Li et al., 2022; Kang and Park, 2024; Wang et al., 2024b). Exercise increases GAD-65, an enzyme for GABA, in spinal cord, strengthening inhibitory control and aiding recovery (Bilchak et al., 2021b). Boosting GABA production helps restore motor function after spinal cord injury (Li et al., 2022).

Traditional treatments for post-traumatic stress disorder and traumatic brain injuries often have limited success. It has led to new arts-based recovery therapies. These therapies use creative activities in structured mental health programs, aiming to promote healing through self-expression and aesthetics (Levy et al., 2025). In China, demand for rehabilitation services has grown sharply in recent decades, mostly due to an aging population. By 2034, about 40% of people may need restorative care, up from 33% in 2019. The increase is especially noticeable among women and varies with age, highlighting the need for personalized recovery plans (Tian et al., 2025).

#### Exercise effects on microglia and immune response

Exercise is widely seen as a safe, effective way to reduce neurological symptoms. Many studies show that physical activity can lessen nerve pain by influencing microglia in the spinal cord and maintaining their balanced states. Over the past decades, exercise effects on the immune system, especially in autoimmune and chronic conditions, have been studied extensively (Chow et al., 2022). Evidence from live models indicates that exercise triggers autophagy via the brain-derived neurotrophic factor/Akt/mammalian target of Rapamycin (mTOR) pathway, helping microglia switch from a pro-inflammatory M1 to an anti-inflammatory M2 type. The switch is key to easing nerve pain and shows promise for treating neuroinflammation (Wang et al., 2024b).

Exercise also boosts immune cell movement in the blood and improves immune surveillance (Brummer et al., 2023). In rats with spinal injuries, 12 weeks of treadmill walking reduced lesion size and macrophage infiltration more than no exercise (Davaa et al., 2021). In another study, 4 weeks of exercise lowered macrophage numbers in the dorsal root ganglion below the injury, with some pain improvements (Chhaya et al., 2019). Although macrophages and microglia had less activity after exercise, the M1 to M2 macrophage ratio and IL-10 levels (linked to M2) stayed stability (Davaa et al., 2021).

Overall, these results show that exercise influences neuroplasticity and immune responses, aiding recovery after CNS injuries. Studying these mechanisms can improve our understanding of how physical activity may serve as an effective therapy for neurological problems.

### Adjusting immune cells to balance brain excitation and inhibition

Outside the CNS, the immune system affects nerve healing. Improving immune responses may help treatment (Tang et al., 2022). CD4^+^ T cells are important for neural repair. Early studies using these cells show promise for clinical use (Gao et al., 2024). For example, engineered T cells that produce IL-10, injected after stroke, changed microglia toward a regenerative state. It shows T cell-microglia interactions are key, opening new immune treatments for stroke and neuroinflammation (Benakis et al., 2022).

Inflammation plays a role in many neurological diseases. Targeting pathways involving microglia, Toll-like receptors, T cells, and cytokine IL-6 may lead to new therapies. Microglia detect danger signals via Toll-like receptor 4, acting quickly. Drugs activating Toll-like receptor 4 show potential. Blocking T cell activity with drugs like Teriflunomide or inhibiting IL-6 with Satralizumab might reduce chronic inflammation (Hanafy and Jovin, 2024).

Microglia also control synapses and the excitation-inhibition balance in brain, which is vital for proper function (Favuzzi et al., 2021). Problems here link to autism, seizures, and neurodegeneration (Henstridge et al., 2019). A recent study finds specific microglial types that affect GABA signaling, offering therapy targets to restore excitatory-inhibitory balance (Favuzzi et al., 2021). Removing microglia after tau injection reduced tau spread and inflammation, indicating the role of microglia in neurodegeneration (Asai et al., 2015). Targeting these cells may slow disease progression (Asai et al., 2015; Favuzzi et al., 2021).

Microglia also shape GABA synapses by forming, removing, or pruning them. Microglial pro-brain-derived neurotrophic factor influences GABA receptors by activating proteins like p75 neurotrophin receptors, reducing GABA_A_R expression. Brain-derived neurotrophic factor from microglia can also activate tropomyosin receptor kinase B, causing degradation of gephyrin, important for GABA_A_Rs, and lowering KCC2. These interactions are critical for understanding neurodegeneration and therapy development (Cramer et al., 2022).

After severe spinal cord injury, the autonomic circuits of spinal cord undergo major changes. Normal stimuli can trigger excessive sympathetic reflexes, causing complications and organ damage. A recent study shows microglia often cluster with active glutamatergic neurons, helping form excitatory synapses across spinal segments (Brennan et al., 2024). It leads to larger sympathetic networks affecting immune, endocrine, and cardiovascular functions (Brennan et al., 2024). Removing microglia during key reorganization phases prevented harmful synapse growth and structural changes. It reduced episodes of autonomic dysreflexia and prevented spinal cord injury–associated immunodepression syndrome. Restoring microglia in mice lacking them fixed circuit structure and function, showing the key role of microglia in autonomic plasticity. Targeting microglia could restore the excitation-inhibition balance, lessening CNS trauma effects (Brennan et al., 2024).

### Nutrition to boost gamma-aminobutyric acid

Diet influences health for a long time. In the 19^th^ century, low-carb diets (around 50 g per day) helped control diabetes (Johnstone et al., 2008; Paoli et al., 2015). The ketogenic diet, low-carbohydrate and high-fat, was used to manage epilepsy. Ancient Greek doctors even used to fast to trigger ketosis and treat seizures (Barzegar et al., 2021). Recently, ketogenic diet has gained attention as an addition or alternative for weight loss and neurological diseases (Grabowska et al., 2024).

Ketogenic diets have many brain benefits: reducing inflammation, lowering oxidative stress, and improving mitochondria (Hallböök et al., 2012; Chinna-Meyyappan et al., 2023). Animal studies support that ketogenic diet protects against epilepsy, cognitive decline, and neurodegeneration (Rusek et al., 2019; Davis et al., 2021; Hersant and Grossberg, 2022). These effects may come from increased brain GABA and lower reactive oxygen species (Calderón et al., 2017). A 2-week ketogenic diet trial raised urinary GABA levels, which may explain its positive effects (Grabowska et al., 2024).

The ketogenic diet may reduce anxiety partly by changing gut bacteria (Attaye et al., 2021), lowering inflammation (Koh S, 2020), and decreasing reactive oxygen species (Tieu et al., 2003; Grabowska et al., 2024). Certain bacteria, like Lactobacillus rhamnosus, produce GABA and influence brain GABA receptors, affecting mood and anxiety (Paoli et al., 2019). These gut-brain interactions are key to how diet can help reduce anxiety. The low-carbohydrate, high-fat diet might also decrease nerve excitability by enhancing GABA via neurosteroids (Hartman et al., 2007; Forte et al., 2016).

To boost GABA, careful diet choices are needed (Molinari et al., 2025). Recent research points to the important role of neurosteroids and GABA receptors, especially within the gut-brain axis (Carver and Reddy, 2013). Since ketogenic diet changes gut bacteria, maintaining a healthy microbiome is vital. Eating fermented foods and prebiotics supports gut health (Włodarczyk et al., 2020). A good gut environment helps strengthen GABA systems and offers neuroprotection. Exploring how diet, gut health, and GABA regulation connect can advance CNS treatments. The approach offers new ways to understand and treat neurological disorders, emphasizing a holistic view of brain health.

## Translational Potential and Limitations

Multiple therapeutic strategies, including KCC2 activation, GABA receptor agonists, cell transplantation, and activity-dependent rehabilitation, have been explored to restore GABAergic tone and rebalance excitatory-inhibitory signaling during both acute and chronic phases of spinal cord injury, traumatic brain injury, ischemic stroke, and neurodegenerative disorders. While preclinical studies have shown progress, and activity-based therapies have demonstrated benefits in improving function after spinal cord injury, significant gaps remain. Notably, a lack of randomized controlled trials and considerable variability among patients’ responses limit their clinical applicability (Jones et al., 2014; Duan et al., 2021).

Transitioning these interventions into routine clinical practice is complicated by the absence of standardized treatment protocols. Personalizing therapy based on injury severity and individual patient needs is paramount to achieving optimal outcomes (Dose and Taccola, 2012; Mazzone et al., 2021). However, overly aggressive or ill-timed GABA enhancement can impair synaptic transmission (Lev-Tov and Pinco, 1992) and suppress spinal circuit excitability, both of which are critical for motor recovery. Achieving a physiological level of inhibition calls for precise control over spatial and temporal patterns, an objective challenged by the variability in treadmill training parameters like intensity and duration during the subacute recovery phase (Shibata et al., 2021). Correlations have been observed between training intensity metrics (e.g., speed and distance traveled) and motor improvement, yet standardized protocols applicable in chronic stage tailored to individual tolerances are still lacking (Yao, 2022; Brennan et al., 2024; Wang et al., 2024b).

Addressing the complex sequelae of CNS injury necessitates an integrated approach that considers both neural and immune dysfunction. While activity-dependent rehabilitation has shown promise, its effectiveness can be limited by injury severity, comorbidities such as cardiovascular issues in traumatic brain injury or frailty in neurodegenerative diseases, and barriers to access. Combining pharmacological agents, like KCC2 activators (e.g., CLP257) or gene therapies targeting GAD-65 or GAD-67, may enhance inhibitory tone and improve recovery prospects across different injury types and stages. The development of adaptive training systems that provide real-time physiological feedback could further refine therapeutic outcomes and warrants further investigation.

In the field of neuroimmune science, engineered T cell therapies have shown promise by reprogramming pro-inflammatory microglia toward a regenerative phenotype, particularly in models of spinal cord injury and stroke. The ketogenic diet also contributes significantly, as it enhances GABAergic signaling partly through modulation of the gut microbiome. Notably, it promotes GABA production via Lactobacillus rhamnosus and concurrently diminishes neuroinflammation. These two approaches may work well together in addressing conditions such as epilepsy, Alzheimer’s disease and mood disorders. Nevertheless, the precise mechanisms governing how GABA signaling interacts with T cell trafficking and microglial polarization in CIDS are still poorly understood. Additionally, further research is needed to clarify how the ketogenic diet influences age-related neurodegeneration.

Achieving a balanced excitatory-inhibitory neural profile requires detailed mechanistic understanding. Combination therapies have demonstrated encouraging results; for example, pairing KCC2 activators with treadmill training in spinal cord injury models increase chloride ionic transporter expression and encourages synaptic plasticity, leading to improved functions (Gomes-Osman et al., 2016; Côté et al., 2017). During stroke recovery, interventions such as baclofen or transcranial magnetic stimulation have been used to reduce spasticity and promote neurovascular repair. Tailoring these strategies to the specifics of injury, such as combining GABA receptor agonists with immunosuppressants in multiple sclerosis, can further refine treatment efficacy.

Despite these advances, challenges remain in translating preclinical successes into clinical applications. Long-term studies are essential to define standardized exercise regimens, especially when adapting high-intensity interval training for various severities of spinal cord injury and understanding dose-response relationships. Interventions with spatiotemporal precision, such as optogenetic modulation of KCC2, could minimize unwanted effects and preserve circuit-specific plasticity. Future investigations should systematically consider variables like injury model, intervention timing, exercise modality, and lesion location (Bilchak et al., 2021a). Such comprehensive research could break the cycle of disinhibition, immune dysfunction, and secondary neurodegeneration, ultimately leading to groundbreaking clinical improvements for individuals with CNS injuries.

## Conclusion and Future Directions

**Figure 4:**
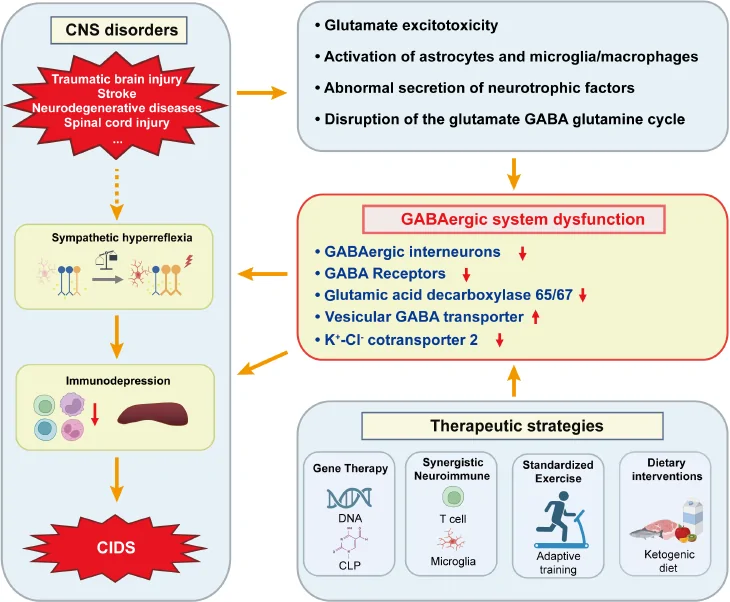
Mechanisms and therapeutic approaches targeting GABA dysfunction in CNS disorders. Trauma-related injuries to the CNS, including traumatic brain injury, stroke, neurodegenerative conditions, and spinal cord injury, disrupt autonomic networks and impair GABAergic signaling. Typical molecular alterations involve the loss of GABAergic interneurons, diminished expression of GABA receptors, decreased production of GAD-65 or GAD-67 enzymes, and a reduction in KCC2. Interestingly, the levels of vGAT tend to increase in some cases. These molecular disturbances interfere with neural plasticity, leading to maladaptive circuit remodeling and playing a role in the development of CIDS. Recent therapeutic strategies aim to re-establish GABA function with high spatial and temporal specificity. Techniques like optogenetic modulation to influence KCC2 activity, alongside gene therapy approaches to boost GAD-65 or GAD-67 expression, are under investigation. Combining immunomodulatory treatments, such as T cell therapies, with GABA receptor agonists and microglial regulation presents a promising avenue to reduce neuroinflammation and restore inhibitory signaling. Rehabilitation interventions emphasize injury-specific exercise regimens and adaptable training programs designed to promote recovery. Additionally, dietary approaches, such as ketogenic diets, have shown potential for influencing GABA pathways and aiding neural repair. Successfully addressing these complex disruptions caused by CNS trauma requires integrated, targeted treatments aimed at restoring inhibitory balance and preventing the progression of CIDS. CIDS: Central nervous system injury-associated immunodepression syndrome; Cl^–^: chloride ion; CLP: chloride ionic load protector; CNS: central nervous system; GABA: gamma-aminobutyric acid; GAD: glutamic acid decarboxylase; K^+^: potassium ion; KCC2: potassium (K^+^)-chloride (Cl^–^) cotransporter 2; NKCC1: Na^+^-K^+^-2Cl^–^-cotransporter 1; vGAT: vesicular GABA transporter.

This review emphasizes how GABA dysfunction acts as a key driver in the cascade that links CNS injury to immunodepression and neurodegeneration. A decrease in GABA-mediated inhibition hampers autonomic regulation, often resulting in excessive sympathetic activity. Such neuroimmune dysregulation manifests as lymphopenia, cytokine imbalance, and heightened infection risk, creating a vicious cycle of neuroinflammation and secondary tissue damage, a central feature of CIDS (**Figure 4**).

Impairments in the GABA system are common across many neurological and psychiatric conditions, including epilepsy, autism spectrum disorder, schizophrenia, and depression (Kaila et al., 2014; Ben-Ari, 2017). Its influence extends to issues like spasticity, neuropathic pain, and immune dysregulation, with evidences from preclinical and clinical studies confirming the pivotal role of GABA in these pathologies (Boulenguez et al., 2010; Sanchez-Brualla et al., 2018; Mapplebeck et al., 2019; Li et al., 2020b, 2022; Bilchak et al., 2021b). Restoring GABAergic function through precisely targeted interventions, while maintaining synaptic plasticity, has thus become a key therapeutic goal (Gong et al., 2021; Lam et al., 2023).

Achieving GABA tone restoration after spinal cord injury involves addressing a range of complex processes. These include the loss of descending inhibitory control, phenotypic changes in primary afferent fibers, depletion of GABAergic interneurons, chloride ionic imbalance, and paradoxical receptor responses (Russ et al., 2013). Recognizing these interconnected mechanisms emphasizes the need for multi-modal treatment approaches. Pharmacological agents such as KCC2 activators, like CLP257, along with gene therapies targeting chloride ionic regulation, have shown promise in reducing symptoms of chronic dysautonomia (Beverungen et al., 2020; Bilchak et al., 2021a). Non-drug strategies, including exercise, can enhance GAD-65 or GAD-67 and KCC2 expression, while reducing vesicular GABA transporter levels, thus bolstering inhibitory signaling and aiding functional recovery (Li et al., 2022; Talifu et al., 2022).

The ketogenic diet promotes a state of nutritional ketosis, which supports GABAergic signaling by increasing brain GABA levels, modulating gut microbiota, particularly via GABA production by Lactobacillus rhamnosus, and activating neurosteroid receptors. Additionally, ketogenic diet reduces neuroinflammation and oxidative stress, demonstrating benefits in epilepsy, neurodegeneration, and anxiety disorders (Tieu et al., 2003; Koh et al., 2020; Attaye et al., 2021; Grabowska et al., 2024). Its capacity to influence T cell-microglial interactions and regulate the gut-brain axis further enhances GABA synthesis and systemic immune responses.

Immunomodulatory strategies, such as engineered CD4^+^ T cells to express IL-10, aim to reprogram microglia toward a regenerative state, thereby decreasing neuroinflammation and supporting axonal repair (Tang et al., 2022). When combined with GABA receptor agonists like baclofen, these approaches can produce synergistic effect, restore autonomic functions of lymphoid organs and foster a bidirectional connection between the CNS and immune system. Fine-tuning the timing and dosing of such interventions is vital to minimize the risk of unintended immunodepression (Tang et al., 2022).

Developing targeted therapies, like optogenetic techniques to modulate KCC2 activity and adeno-associated virus-based gene delivery of GAD-65 or GAD-67, is vital for expanding treatment options for CNS injuries. Challenges remain in establishing efficient delivery systems and balancing inhibitory modulation with neuroregeneration goals (Gong et al., 2021; Mazzone et al., 2021; Brennan et al., 2024). Consistency in exercise protocols for spinal cord injury is also crucial; the variability in intensity and duration across preclinical studies hampers progress toward clinical application (Shibata et al., 2021). High-intensity interval training, for example, shows potential for stimulating neurovascular remodeling and increasing KCC2 expression. However, understanding how these responses vary with age and injury severity is essential for optimizing such interventions effectively.

To fully capitalize on the combined effects of different treatments, integrated models that incorporate GABAergic signaling, immune responses, and motor plasticity are needed. Future research should focus on developing precise, spatiotemporal methods to restore GABA activity, methods that reduce disinhibition while supporting activity-dependent neural recovery. Additionally, exploring the interactions between microglia and T cells could provide clearer insights into targeted therapies for CIDS. Combining pharmacological agents, behavioral training, and dietary modifications may overcome the limitations inherent in single-modality approaches.

Sympathetic hyperreflexia, often observed in spinal cord injury–associated immunodepression syndrome and CIDS, results largely from GABA system deficits. Currently, strategies to restore the excitation-inhibition balance, such as modulating ionic channel plasticity, targeting microglia, or using activity-based therapies, offer promising avenues for reversing immunodepression. Studying the mechanisms underlying spinal cord injury-associated immunodepression syndrome and CIDS may reveal broader insights into the pathophysiology of CNS injuries and identify the GABA system as a critical therapeutic target.

## Data Availability

*Not available.*
